# Research and development of new tuberculosis vaccines: a review

**DOI:** 10.12688/f1000research.16521.2

**Published:** 2019-02-24

**Authors:** Lewis K. Schrager, Rebecca C. Harris, Johan Vekemans

**Affiliations:** 1 Consultant, World Health Organization, Geneva, Switzerland; 2 London School of Hygiene & Tropical Medicine, London, UK; 3 Initiative for Vaccine Research, World Health Organization, Geneva, Switzerland

**Keywords:** Tuberculosis, Mycobacterium tuberculosis, vaccines, immunization

## Abstract

Tuberculosis kills more people worldwide than any other single infectious disease agent, a threat made more dire by the spread of drug-resistant strains of *Mycobacterium tuberculosis (Mtb)*. Development of new vaccines capable of preventing TB disease and new *Mtb* infection are an essential component of the strategy to combat the TB epidemic. Accordingly, the WHO considers the development of new TB vaccines a major public health priority. In October 2017, the WHO convened a consultation with global leaders in the TB vaccine development field to emphasize the WHO commitment to this effort and to facilitate creative approaches to the discovery and development of TB vaccine candidates. This review summarizes the presentations at this consultation, updated with scientific literature references, and includes discussions of the public health need for a TB vaccine; the status of efforts to develop vaccines to replace or potentiate BCG in infants and develop new TB vaccines for adolescents and adults; strategies being employed to diversify vaccine platforms; and new animal models being developed to facilitate TB vaccine development. A perspective on the status of these efforts from the major funders and organizational contributors also is included. This presentation highlights the extraordinary progress being made to develop new TB vaccines and provided a clear picture of the exciting development pathways that are being explored.

## Table of Contents

**1. Public health need for new tuberculosis vaccines and landscape analysis**
a. Introduction
b. The WHO Global TB Programme
c. The Value Proposition for TB Vaccines
d. Overview of the tuberculosis vaccine clinical pipeline
e. Modeling impact according to vaccine profile
**2. Vaccines for BCG replacement**
a. The BCG vaccine’s profile and role in public health
b. VPM1002
c. MTBVAC
**3. Vaccines for adolescents and adults (phase 2 and beyond)**
a. VPM1002
b. M72/AS01E
c. H56:IC31
d. ID93/GLA-SE
e. RUTI™ for Adjunctive Immunotherapy
f. Vaccae™
**4. Diversifying TB vaccine platforms**
a. Importance of immune characterization
b. Cytomegalovirus (CMV) recombinant vaccines against TB
c. Novel emerging vaccine technologies: mRNA-based vaccines
d. New candidates for live attenuated TB vaccines
e. A systematic antigen discovery approach in humans
f. Antibody-generating vaccines for TB
g. Diverse T-Cell responses against *Mtb*
h. Immune correlates and signatures of protection
**5. Use of models in translational TB vaccine research**
a. Small animal models
b. The role of a continuous exposure natural transmission animal model for vaccine development
c. Simian models of TB
d. Achieving sterilizing immunity in animal challenge models
e. Mucosal immunisation against TB
f. Alternative routes of BCG immunization
g. Progress in clinical use of a controlled human infection model (CHIM) for vaccine testing
**6. Perspectives from funders and global health organizations working on TB vaccines**
a. Perspectives from the Bill and Melinda Gates Foundation
b. The Global TB Vaccine Partnership
c. Aeras
d. European and Developing Countries Clinical Trials Partnership
e. The Stop TB Partnership, working group on new TB vaccines
f. The Tuberculosis Vaccine Initiative
g. Proposed stage gate criteria for TB vaccines
**7. Conclusions**

## 1. Public health need for new tuberculosis vaccines and landscape analysis

### a. Introduction

The World Health Organization (WHO) provides guidance on priority targets and development pathways for vaccines against diseases of high public health interest. The involvement of the WHO in vaccine development is driven by assessments of medical need and technical feasibility, and an absence of market incentives and sufficient funding to adequately drive the development process. As tuberculosis (TB) kills more people globally than any other single infectious agent ^1^, developing a TB vaccine ranks among the highest global health priorities from a medical needs perspective. A number of factors support the technical feasibility of a TB vaccine, including the fact that an estimated 90% of persons infected with *Mycobacterium tuberculosis* ( *Mtb*) do not progress to active disease ^1^, evidence suggesting that past *Mtb* infection provides some protection against new infections ^2, 3^, and the existence of a century-old vaccine, Bacillus Calmette-Guérin (BCG), that provides partial protection in children which may, under some circumstances, extend for decades ^4^. The current imperative is to improve our understanding of the type of immunological responses needed to provide robust protection against *Mtb* infection or TB disease, and to use this information to efficiently develop new, safe and effective TB vaccines.

In response to the compelling public health need for a TB vaccine, in October 2017 the WHO convened experts and representatives of important stakeholder institutions involved in vaccine development to assist the WHO in a two-pronged effort to accelerate TB vaccine development. The first effort was dedicated to defining a preferred product characteristics (PPC) guidance document for new TB vaccines, now publicly available ^5^. The second was to convene a meeting providing an opportunity for the exchange of cutting-edge information relevant to TB vaccine development, from basic science to later stage research. The proceedings of this meeting are summarized in this document.

### b. The WHO Global TB Programme

Each year, the WHO publishes the Global Tuberculosis Report, documenting the overall status of the global TB epidemic. The 2017 version of this report highlights the following statistics for the state of the TB epidemic in 2016 ^1^:

- 1,674,000 TB deaths, of which 374,000 were in HIV co-infected persons (“the ninth leading cause of death worldwide and the leading cause from a single infectious agent, ranking above HIV/AIDS” ^1^); 374,000 of these deaths occurring in HIV-infected persons
- 10.4 million cases of TB disease, 56% of which were in 5 countries: India, Indonesia, China, the Philippines and Pakistan;
- 600,000 cases of rifampicin-resistant TB (RR-TB), of which 490,000 were multidrug-resistant TB (MDR-TB). Globally, an estimated 4.1% of new cases and 19% of previously treated cases had MDR or RR-TB, with 47% of the global total of drug-resistant cases reported from China, India and the Russian Federation. 250,000 of these 490,000 died.
- A U.S. $2.3 billion funding gap for financing TB care and prevention existed in 2017

The WHO strategy to tackle the global epidemic of TB was set forth in the WHO End TB Strategy, endorsed by the WHO General Assembly in 2014 ^6^. The End TB Strategy relies on a number of priorities in the global response to the TB epidemic. These priorities include improving approaches to identifying cases of TB, addressing MDR-TB as a true public health crisis, and accelerating strategies to better prevent, identify and treat TB in HIV-infected individuals. A three-pillared approach to the TB epidemic is being implemented: 1) to advocate for integrated, patient-centered TB care and prevention; 2) to support the creation of bold policies and supportive systems to curb the spread of TB; and 3) to support intensive research and innovation to create the new tools needed to prevent and treat control TB, including better diagnostics and drugs, and a new vaccine capable of preventing TB disease.

A key objective of the WHO End TB Strategy is a 90% reduction in TB incidence, from the 2015 annual incidence of greater than 100 cases per 100,000 population to less than 10 cases per 100,000 persons by 2035 ^6^. Currently, the annual decline in TB incidence is not nearly fast enough to meet the expressed goals ^1^. As evidenced by the successes of the social protections provided under the Marshall Plan in the aftermath of World War II, a 10% rate of decline could be considered feasible through optimization of currently available social and medical interventions, although this is a major proposition in and of itself which would require a heightened degree of global commitment to achieve. Without the development of new TB prevention modalities, however, this rate would be expected to flatten out to a 5% decline per year by 2025, again, not nearly sufficient to meet the End TB Strategy 2035 goals ^6^. Only the introduction of new tools, including better point-of-care diagnostics capable of quickly and reliably diagnosing TB, including cases of drug-resistant (DR) TB; better drugs for treating drug-sensitive and DR-TB over a shorter period of time than is currently required; and, most importantly, a new vaccine capable of preventing TB disease, introduced by 2025, would result in an acceleration to the 17% per year decline of TB incidence necessary to meet the 2035 goals ^6^.

Currently, a U.S. $2.3 billion annual research gap exists in efforts to develop new tools to control TB, including better diagnostics and drugs, as well as TB vaccines ^1^ a figure that may represent a conservative estimate of the actual funding shortfall, particularly considering the need to invest in efforts that will result in the development of an effective vaccine by 2025 for the targets to be reached. The ongoing United Nations (UN)–led efforts to prioritize action against TB is illustrated by a series of high-level political meetings. Top-level political engagement was demonstrated in Russia in November 2017 at the gathering of 194 ministers of health, research and finance, alongside the WHO Director General and the President of the UN General Assembly. This was followed by an interactive civil society meeting, attended by more than 250 representatives from civil society, academia, NGOs, and the private sector, setting the stage for the first ever UN General Assembly high level meeting (HLM) on TB in New York in September 2018. The HLM aims to deliver an ambitious political declaration on TB endorsed by Heads of State, which will strengthen action and investments to end TB. Issues to be discussed include the increase in drug-resistant TB strains, the need to provide adequate resources to control the epidemic, and to create a universal monitoring program to track the epidemic and the world’s response to it, will be highlighted. Accelerating development of a vaccine for TB will be a critical component in the future of TB prevention and care, therefore urgently requires increased resource allocation and political support in upcoming years.

### c. The value proposition for TB vaccines

A key question that arises when attempting to assess the value of TB vaccines is how to measure “value”. Possible measures of value include the payer perspective – savings in direct healthcare costs resulting from the protective effect of vaccines; a human perspective – assessing reductions in death and disability; and a societal perspective – determining the overall benefits to society that result from vaccine-induced protection. Different stakeholders will have different views on which of these approaches best defines the value of vaccines. Perspectives of vaccine value also will differ based on whether the value proposition involves a regional, countrywide, or global assessment.

A model of the health impact of the introduction in 2024 in low and middle income countries of a 60% efficacious vaccine with 10 years protection delivered to adolescents and adults, with coverage equal to school attendance for routine vaccination of 10 year olds and 72–76% for periodic mass campaigns, suggests that approximately 17 million TB cases could be averted between 2024 and 2050 ^7^. If such vaccine were to be administered to infants, with approximately 90% coverage rate, 890,000 TB cases would be cumulatively averted by 2050. These benefits need to be weighed against the total systems cost of vaccine administration, including the purchase price and the cost of administration, transportation and storage, national program management and the facilities and equipment necessary to deliver and administer a vaccine. In the modelled scenarios above, the median cost per disability-adjusted life year (DALY) averted in low or middle income settings ranged from cost saving to $720 per DALY averted in the adult/adolescent scenario, and $1,690–$18,110 per DALY averted in the infant scenario ^7^. Issues faced in the early stages of vaccine development, such as efficacy and duration of protection, and issues arising later, such as vaccine price, regimen of administration, thermostability and availability (overall supply) all contribute to the cost of vaccine administration and should be taken into consideration at the very earliest stages of the development of a vaccine.

### d. Overview of the TB vaccine clinical pipeline

Currently, multiple vaccine candidates are being assessed in clinical trials. Vaccine strategies being assessed include live, attenuated mycobacteria (MTBVAC, VPM-1002); killed, whole cell mycobacteria (DAR-901, M. vaccae, MIP), mycobacterial extracts (RUTI); adjuvanted protein vaccines (M72/AS01E, H4:IC31 (discontinued), H56:IC31, ID93 + GLA-SE), and viral vectored vaccines (Ad5Ag85A, ChAdOx185A/MVA85A, TB/FLU-04L). Although the target populations in these trials include adolescents, adults and infants, it is generally recognized that vaccinating adolescents and adults represents the most efficient means of stopping the cycle of *Mtb* transmission and thereby offers the best opportunity to control the global spread of *Mtb*, even to infants and children ^7, 8^.

Four potential indications have been identified as targets for TB vaccine development in adolescents and adults: 1) prevention of TB disease (PoD); 2) prevention of recurrent TB disease, which can be related to relapse or re-infection, in persons cured post-treatment for active disease (PoR); 3) prevention of established *Mtb* infection in persons uninfected with *Mtb* (PoI); and 4) as an immunotherapeutic adjunct to drug treatment to shorten curative regimens or increase the efficacy of treatment of drug-resistant strains. In infants, the major goals have been to develop a vaccine that is more efficacious and/or safer than BCG that could serve as a BCG replacement and to develop a booster to improve and extend the protection provided by BCG.

PoD represents the highest priority indication, as modeling suggests that preventing TB disease in adolescents and adults would be the quickest way to control the global TB epidemic ^7, 8^. The major issue in developing a vaccine for a PoD indication, however, is the cost of conducting late-stage efficacy trials given the relatively large sample sizes and prolonged period of post-vaccination follow-up required to accrue adequate endpoints in the general, healthy population. In light of this, novel trial designs for phase 2 proof of concept studies have been developed to reduce the risk of failure in expensive late-stage trials. These novel designs have focused on assessing candidate TB vaccines for clinically meaningful biological effects in selected high-risk populations, to allow shorter, less expensive trials for assessment of efficacy.

One such novel design is based on prevention of recurrent TB disease in recently cured TB patients since this population is at an elevated risk of developing TB compared to individuals that have never had TB, due either to reinfection or to reactivation of existing infection. While a PoR endpoint could serve as a licensable indication in its own right, it currently is also being assessed as a possible means of de-risking a decision to move a vaccine candidate forward into late-stage PoD evaluations in the general population ^9, 10^.

Assessing the ability of a candidate vaccine to prevent establishment of *de novo Mtb* infection represents another strategy to demonstrate a clinically meaningful biological effect. If populations experiencing a high force of *Mtb* infection were recruited, such as adolescents in the Western Cape of South Africa, a PoI endpoint trial could potentially be less expensive to conduct than a PoR trial ^11^. Results from a PoI trial of the adjuvanted protein vaccine, H4:IC31 versus BCG revaccination, conducted in adolescents in the Western Cape were recently published (after the meeting reported here). This trial demonstrated that neither the H4:IC31 vaccine nor revaccination with the BCG vaccine prevented initial quantiferon (QFT) conversion (efficacy point estimates of 9.4%, P=0.63 for H4:IC31; 20.1%, P=0.29 for BCG). BCG revaccination, however, reduced the rate of sustained QFT conversion (efficacy 45.4%, P=0.03); efficacy of sustained QFT conversion for H4:IC31 was 30.5%, P=0.16) ^12^. The implications of these results for BCG revaccination strategies are currently being discussed. A recent review of available data on BCG revaccination before these data were released led WHO to conclude that the existing evidence, prior to the completion of the BCG revaccination PoI trial did not support BCG revaccination policy ^13^.

Unlike a PoR endpoint, however, it is unclear whether a PoI endpoint could serve as an indication for licensing for two reasons: 1) only approximately 10% of *Mtb*-infected individuals ultimately develop active TB, so it is theoretically possible that infection prevention, should it be demonstrated, is only occurring in persons who would otherwise control their *Mtb* even without vaccination and not develop TB disease; and 2) the prevention of infection endpoint depends on assessments such as the tuberculin skin test (TST) or interferon-gamma release assay (IGRA) whose specificity and sensitivity are not 100%.

Both the PoI and PoR approaches pose a risk when used as experimental endpoints towards the development of a PoD vaccine, as it remains unknown whether these other endpoints actually predict efficacy for PoD in the general population. It is possible that preventing disease, preventing infection and preventing recurrence of disease in cured patients may require fundamentally different immunological responses, thereby leading to positive or negative results that would not be predictive of a positive PoD outcome.

The current moment represents a critical juncture for TB vaccine development. In 2018–2020, clinical efficacy data are scheduled to be released from five phase 2b proof of concept studies: Vaccae, H4/BCG revaccination (data subsequently released and described above), M72/AS01 (data subsequently released and described below), Dar901 and VPM1002. Results from these trials will paint a clearer picture as to the potential of the global vaccine candidate pipeline, optimal paths for future TB vaccine design and development strategies, and the predictive ability of current animal models. Ideally, candidates that demonstrate efficacy will also enable discovery of candidate correlates of protection, with potential to substantially streamline further TB vaccine development.

Many challenges must be addressed to develop a successful TB vaccine. *Mtb* represents a complex pathogen that has evolved with humanity for tens of thousands of years, allowing it to evolve in such a way as to survive the normal range of immune responses directed against it. The absence of a known correlate of immune protection against TB in humans has hampered vaccine discovery and development, as has the inability to assess the predictive nature of animal challenge models given the lack of vaccine efficacy data in humans to date. Late stage efficacy trials, particularly phase 3 trials for efficacy and safety, are long and expensive, as are subsequent phase 4 programs, together representing a potential disincentive for biotechnology and pharmaceutical companies to expend resources given the relatively high risk of failure and expectations of limited commercial returns. Moreover, the TB vaccine field remains severely underfunded, particularly given the potential impact of a vaccine in curbing the TB epidemic and the global societal cost of TB, estimated at $19.2 billion in 2017, including a $7.2 billion cost for treatment and control activities and $12 billion indirect costs to the economies ^1^.

Despite these challenges, however, it is becoming clear that developing new TB vaccines is an achievable goal. The partially protective effect of BCG, capable of preventing up to 80% of cases of severe, disseminated TB in infants and young children, provides important supportive evidence of this point. Additional evidence comes from the 90% of *Mtb*-infected persons who are capable of controlling their infection throughout their lives without developing disease; and the intriguing examples of individuals at high risk of *Mtb* infection who, when assessed for *Mtb* infection by TST or IGRA, demonstrate transient positivity on repeated assessments before reverting to a consistently negative state, a situation with unclear clinical significance but which has been interpreted by some as evidence of early immunological containment of *Mtb* infection and a reduced risk of TB disease ^14– 17^. Examples of individuals with lifelong exposure to *Mtb*, through household or occupational contact, for example, who never convert their TSTs or IGRAs, may represent another example of natural immunity to *Mtb* that a vaccine could be able to replicate or improve upon ^18^.

Accordingly, there are reasons for optimism in TB vaccine development. New strategic directions include diversifying the pipeline to explore candidates that generate immunity beyond CD4+ Th1 T-cells, including non-conventional, cellular immunity and trained innate immunity ^19^; assessing new routes of vaccine administration, including aerosol and, possibly, intravenous approaches; investigating the extent to which antibody generating vaccines may contribute to protection afforded by the current, cell-mediated immune generating candidates; and utilizing new tools for vaccine candidate R&D, including positron emission tomography-computerized tomography (PET-CT) scans ^20^ and bar-coded *Mtb* strains ^21^ permitting ultrasensitive assessments of animal challenge models, and a potential controlled human infection model (CHIM) for *Mtb* ^22, 23^. These new strategic directions in TB vaccine development add to the sense of optimism and excitement in the field.

### e. Modeling impact according to vaccine profile

Mathematical modeling techniques provide useful estimates of the future impact of vaccines in development. A review of twenty-four mathematical modeling studies applied to the case of TB vaccine development and implementation, provide insights relevant to decisions concerning the optimum age of vaccination, the most impactful vaccine indication, and the minimum duration of vaccine effect, efficacy levels and implementation strategy needed to have a meaningful impact on the TB epidemic ^8^.

Mathematical models suggest that, in low- and middle-income countries overall (LMICs), targeting adolescents and adults for TB vaccination campaigns will result in a greater and faster impact on the TB epidemic before 2050, than would targeting infants ^7^. In aging, reactivation driven, epidemics like China, a high proportion of TB disease occurs in older adults; accordingly, targeting adolescents and younger adults would not be expected to have an important effect in controlling TB before 2050, as compared to a vaccine with administration targeted to older adults.

Vaccinating infants with an improved TB vaccine would represent an important public health benefit over the long term but would not have as great an impact before 2050 as would an adolescent/adult vaccine, mainly because infants and young children are not an important source of *Mtb* spread. Accordingly, the public health impact of an infant vaccine would be delayed until adolescence, when the burden of *Mtb* infection, and the possibility of spreading *Mtb* to others, increases. Moreover, an infant vaccine would need to demonstrate higher efficacy and/or longer durations or protection than a vaccine administered to adolescents and adults to have an important, and cost effective, effect on public health ^7^. Mathematical modeling of the impact of vaccinating infants extends to the year 2050. Beyond that, there is too much uncertainty to draw conclusions with confidence.

Modeling suggests that, before 2050, a PoD vaccine would have a greater and faster effect on controlling *Mtb* spread than would a PoI vaccine ^8^. Even if a 100% efficacious PoI vaccine were to be developed and made available by 2025, the target date for TB vaccine introduction set by the WHO End TB Strategy, a PoD vaccine also would need to be utilized if the 2035 End TB Strategy targets were to be met. The body of available mathematical modelling literature is at equipoise as to whether the effect of a PoD vaccine would be greater if administered to persons who are not yet infected with *Mtb* as compared to those who are latently infected, with three models predicting that a pre-infection vaccine would provide the greatest impact, while four suggest that a post-infection vaccine would have a greater effect on TB control. More recent modelling evidence, specifically exploring this issue suggests that in aging, reactivation driven epidemics like China, because over the modelled vaccination time frame most TB disease is predicted to occur among the elderly population, a PoD vaccine administered to persons latently infected with *Mtb* would have greatest and most rapid impact ^24^.

While a PoR vaccine would be expected to contribute to TB control efforts, no mathematical modeling of the public health effect specifically of a PoR vaccine as yet has been undertaken.

Mathematical modeling exercises reinforce the intuitive conclusion that a vaccine with greater efficacy and a longer duration of effect will have greater benefit than those with lesser degrees of efficacy and durations. In addition, modeling has suggested that a PoD vaccine with protective efficacy as low as 20%, and duration as short as 5 years, administered to adolescents and adults, could have an important and cost-effective effect in controlling the spread of *Mtb* in LMICs ^7^. Vaccines with shorter duration of effectiveness could be compensated for by higher efficacy or performing more frequent mass vaccination campaigns, to the extent that resources and logistical realities might permit.

In summary, mathematical models of TB vaccine effect suggest that, for maximum impact before 2050, the optimal new TB vaccine would prevent *Mtb* disease (although in transmission-driven epidemics a vaccine preventing infection would be useful), would be targeted for use in adolescents and adults, and would have a duration of effect of at least five years. Modeling also emphasizes that optimal vaccine strategies to control TB may differ between countries based upon the segment of the population that is driving the epidemic and so should be tailored accordingly. Future modeling exercises are needed to explore the effect of a PoR vaccine, to get a clearer picture of optimum vaccine strategies in other high burden countries such as India and South Africa, to assess the impact and cost-effectiveness of vaccines on controlling the spread of multi-drug resistant TB (MDR-TB) and extensively drug resistant TB (XDR-TB), to gauge the impact of new vaccines in combination with the scale up of other interventions, and to assess the impact of TB vaccine administered as immunotherapeutic agents in persons being treated for active TB.

## 2. Vaccines for BCG replacement

### a. The BCG vaccine’s profile and role in public health

Optimally, certain information should be known when attempting to develop a vaccine, such as the immune mechanisms necessary and sufficient to protect against infection and/or disease, the antigens that stimulate a protective immunological response, the antigen delivery platforms best capable of evoking a protective response in a safe and acceptable manner, and confirmation of the protective mechanisms in human clinical efficacy studies. Currently, none of this information is available for TB vaccines. Accordingly, additional information must be sought and utilized to justify and guide the TB vaccine development effort.

There is some skepticism about whether an effective vaccine can be developed against TB. We know that about 90% of people infected do not develop disease and that individuals who are immunocompromised have 10-fold greater risk, establishing the importance of protective immune responses. One interesting piece of information comes from an observational clinical study published in the mid-1930s ^2^. In this study, nurses entering service in a TB hospital in Norway were assessed for their rate of TB based on their TST status prior to entering this service. This study revealed that nurses who were TST positive had a 96% reduced risk of developing clinical TB than did the nurses who were TST negative, with an annual TB rate of 4% to 5% observed in the TST-negative nurses, suggesting a meaningful degree of protection against TB disease stemming from latent TB infection (LTBI). A later analysis of 14 studies confirmed this result, with LTBI found to be 78% protective against TB disease, even though *Mtb* infection, in itself, poses a risk for developing TB ^3^.

The bacille Calmette-Guérin (BCG) vaccine represents the only successful vaccine as yet developed for TB. BCG is derived from *Mycobacterium bovis*, a bovine mycobacterium passaged 230 times in the Pasteur laboratory in Lyon by Drs. Calmette and Guérin between 1908 and its first human use in 1921. It has since become the most widely used vaccine in human history, with well over 3 billion doses administered; currently, more than 100 million children annually are administered BCG within hours or days of birth, representing 89% of the annual global birth cohort. BCG provides excellent protection in infants against disseminated miliary and meningeal TB, particularly serious forms of the disease with high mortality rate (RR 0.1; 95% CI 0.01 - 0.77) ^25^.

While evidence of BCG-induced protection against miliary and meningeal TB in infants is widely accepted, the degree to which BCG prevents pulmonary TB in infants, children and adults remains controversial. In the past, some of the differing results has been attributed to the variety of BCG strains currently being used globally, yet in meta-analyses of clinical trials utilizing different BCG strains, no statistically significant difference in efficacy has been identified ^25^. An observational study determined that LTBI provides better protection than BCG vaccination against developing pulmonary TB in adults ^2^, while a meta-analysis of randomized clinical trials of BCG efficacy in children with known exposure to *Mtb* identified a protective effect against acquiring *Mtb* infection of 27% (RR 0.73, 95% CI 0.61 – 0.87) and a protective effect against developing active TB of 71% (RR 0.29; 95% CI 0.15 – 0.58) ^26^.

Overall, the results of studies of BCG effectiveness, beyond prevention of miliary and meningeal TB in infants, has been marked by extraordinary variability and inconsistency, with rates of BCG-induced protection varying from 80% to totally non-protective at any age group. A number of hypotheses have been advanced to explain this variation and relative lack of effectiveness ^27^. These include differences in key antigens between *Mtb* and BCG, including the absence in BCG of the important *Mtb* RD1 and ESX genetic regions; differences in the potency of the various BCG strains in use globally; and interference from exposure to atypical environmental mycobacteria. Mycobacterial exposure, generally in countries closer to the equator, has the potential to confound assessing the efficacy of BCG or any new vaccine candidate in populations whose immune systems have been engaged by cross-reacting mycobacterial antigens. Another unknown about BCG is the extent to which the vaccine persists following infant vaccination.

BCG administration has changed since its first delivery to an infant whose mother had active TB in 1921. Initially, BCG was administered orally, where most organisms were presumed to be killed by stomach acid. Calmette, however, observed bacillemia following oral administration, leading later to a switch of BCG administration to the intradermal (ID) route. Experiments with intravenous (IV) administration of BCG to rhesus macaques in the early 1970s demonstrated significantly greater protection against pulmonary and hematogenous TB following aerosol *Mtb* challenge than in macaques receiving ID BCG ^28^. Recently, there has been renewed interest in the possibility of greater effectiveness of BCG if administered in a way that permitted broader diffusion in the lungs – via inhalation of an aerosolized preparation of BCG ^29^, or systemic dissemination such as would occur following IV administration ^30^. Preclinical application of such approaches offers the opportunity to understand the immunological mechanisms that convey heightened protection in, for example, *Mtb* challenge models utilizing non-human primates such as rhesus macaques and to explore possible clinical applicability. While early, exploratory clinical trials of inhaled BCG have not identified safety concerns ^31^, assuring that an IV route is sufficiently safe for humans is likely to prove challenging.

While BCG generally is regarded as safe, hematogenous dissemination of 1% following ID administration has reported, mainly in infants with HIV infection and other forms of immunosuppression ^32^. For this reason, BCG is contraindicated for use in immunosuppressed infants. The potential for developing serious complications, including death, following uncontrolled hematogenous dissemination when administered to immunosuppressed infants represents an important rationale driving the need for replacing BCG with a safer TB vaccine that can be administered to all infants, regardless of HIV infection status or immune competence.

Improved understanding of *Mtb* and BCG pathophysiology has led to potential paths forward for improving BCG. As BCG is missing the RD1 and ESX genetic regions present in *Mtb*, one possible future approach is to replace them in genetically modified BCG strains. Additionally, it is known that *Mtb* is capable of escaping the macrophage endosome and thereby has its antigens processed in the cytosol, inducing cytotoxic T-lymphocyte responses, and that antimicrobial peptides derived from cytotoxic lymphocytes (CTL) are able to kill *Mtb* within infected macrophages *in vitro.* While BCG is incapable of escaping the endosomes or generating a significant CTL response, a BCG-derived TB vaccine, VPM1002, has been genetically manipulated to permit its escape from the endosome, resulting in cytosolic antigen processing ^33^. The extent to which this alteration may improve BCG efficacy for use in both infants and adults is under active investigation.

Finally, developers of vaccines targeted at replacing BCG in infants will need to take into consideration the effects of BCG unrelated to protection against TB. BCG vaccination has been demonstrated to provide protection against leprosy, and this constitutes one of the bases for BCG recommendation ^13^. Studies of BCG-associated protection against Buruli ulcer disease, however, have demonstrated mixed results ^34, 35^. Improvement in all-cause mortality in LMICs has also been reported ^36^. Additionally, intravesicular BCG has been demonstrated to provide a significant degree of protection from recurrence of superficial bladder cancer as compared to chemotherapy ^37^. Ultimately, investments will need to be made to support clinical studies to fully evaluate all candidates intended to replace BCG, as well as any new TB vaccine.

### b. VPM1002

VPM1002 is a recombinant BCG (rBCG) vaccine being developed both as a replacement for BCG vaccination in infants and as a TB vaccine in adolescents and adults ^33^. In VPM1002, a listeriolysin gene has been added to the BCG genome and a urease gene has been deleted, permitting the rBCG to escape the macrophage lysosome, which occurs in *Mtb* infection but not with BCG. Processing VPM1002 antigens in the cytosol results in activation of the AIM2 inflammasome, inducing autophagy of the VPM1002-infected macrophage and stimulating innate immunity in a manner not seen with BCG. The manufacturing process for VPM1002 also offers the prospect of avoiding the frequent, global shortages of BCG, as it is manufactured using fermentation media, with a 50-liter batch yielding approximately 5 million doses.

The safety and tolerability of VPM1002 has been assessed in a recently-concluded phase 2 trial of HIV-exposed and HIV-unexposed infants in sub-Saharan Africa (NCT02391415); data from this trial is awaiting public release. A phase 3 trial, comparing VPM1002 safety and efficacy to BCG in 10,000 South African infants is scheduled to begin in 2019, with completion targeted for 2021.

### c. MTBVAC

MTBVAC is the only attenuated vaccine derived from *Mtb* currently in clinical trials. To create this vaccine, two stable deletions were made in the genome of an *Mtb* clinical isolate, selected so as to avoid laboratory subculture: deletions of the *phoP* gene, needed to control the transcription of key *Mtb* virulence genes permitting its survival in host cells; and *fadD*26 gene, required for the synthesis of cell surface lipids that play a critical role in *Mtb* pathogenicity ^38^. As MTBVAC is derived from a clinical *Mtb* strain, and only carries two specific deletions in its genome, the vaccine candidate possesses 519 more epitopes (1603 epitopes) than currently used BCG (1084 epitopes) ^39^. Challenge experiments in mice demonstrated improved protection by MTBVAC as compared to BCG, with protection associated with a T-cell mediated response to two *Mtb*-specific antigens, CFP10 and ESAT6, present in MTBVAC but absent in BCG ^40^.

MTBVAC is primarily being developed to replace BCG as a priming immunization against TB. The rationale for targeting infants includes the need for an improved vaccine over BCG given the remaining burden of pediatric TB, and the opportunity to deliver vaccination to a population without prior sensitization to BCG, *Mtb* or environmental mycobacteria, thereby avoiding potential “masking” or “blocking” effects on MTBVAC-induced protection.

A phase 1a study, conducted in BCG-unvaccinated adults living in an area not endemic for TB, demonstrated the safety and immunogenicity of MTBVAC (NCT02013245) ^41^. MTBVAC currently is being further assessed for safety and immunogenicity in newborns in a phase 1b dose escalation study conducted in a TB-endemic region of South Africa, with a safety arm in adults (NCT02729571). Vaccination of newborns enrolled in this study was completed in September 2016, with one-year follow-up in September 2017. A phase 2a dose-defining safety and immunogenicity study of MTBVAC in 99 HIV-unexposed newborns in TB-endemic regions of sub-Saharan Africa is being planned (NCT03536117). This study is also intended to build capacity for future, later-stage evaluations of this vaccine.

In addition to use in infants, MTBVAC is being assessed for use in adolescents and adults previously vaccinated with BCG. Preclinical studies of revaccination of guinea pigs with MTBVAC have demonstrated improved protection compared to that conferred by BCG ^42^. Initiation of a phase 1b-2a, double blind, randomized, BCG-controlled, dose-escalation safety and immunogenicity study in 120 healthy South African adults, ages 18–50 years, with and without LTBI is being planned (NCT02933281).

## 3. Vaccines for adolescents and adults (phase 2 and beyond)

### a. VPM1002

In addition to its development as a vaccine to replace BCG in infants, VPM1002 also is being developed as a TB vaccine for adolescents and adults. A phase 2b/3, randomized, double-blind, placebo-controlled trial to assess VPM1002 vaccine efficacy in preventing recurrence of TB in adults recently treated and cured of active TB (PoR) is underway in India (NCT03152903). Sample size calculations for this study assume a 5% rate of TB recurrence in the placebo arm and a 50% reduction of TB recurrence among subjects in the VPM1002 arm during a 12-month follow-up period. Two thousand persons (1,000 in each arm) will be enrolled to achieve 80% power at a 5% significance level, with a 10% estimated drop-out rate. A phase 3 study to protect against TB disease among household contacts of persons with active TB also is being planned.

### b. M72/AS01E

M72/AS01E, the GSK proprietary candidate vaccine, consists of a fusion protein expressing two immunogenic *Mtb* antigens: Mtb39A, a membrane-associated protein expressed early in the *Mtb* life cycle, putatively identified as an immune evasion factor; and Mtb32A, a constitutively expressed secreted protein and a putative serine protease, combined with the adjuvant system AS01E containing monophosphoryl lipid A (MPL) and QS21 in a liposomal suspension, geared to promote a Th1 immune response ^43^. Key components of the target product profile (TPP) for M72/AS01E include preventing TB in adults and adolescents; an efficacy level of 70% or greater (no minimally acceptable level of efficacy has yet been set by the developers); a clinically acceptable safety profile, including safety for use in HIV-infected individuals; acceptability for co-administration with widely recommended adolescent vaccines when administered in a 2-dose regimen 1 to 6 months apart; and a duration of protection of 10 or more years. The possible need for boosters will be explored.

The M72/AS01E candidate vaccine was first introduced into humans in 2004; clinical development has been conducted in collaboration with Aeras. M72/AS01E has been tested for safety and immunogenicity in 12 completed phase 1 and phase 2 studies. The vaccine has been tested in adults who were PPD negative, PPD positive ^44^, HIV negative ^45^, HIV positive on antiretroviral therapy (ART) ^46^, and HIV positive not receiving ART ^45^. Adults during or after TB treatment were the focus of one phase 2 trial. Adult trials have been conducted in non-endemic and endemic TB settings. The vaccine has also been assessed in adolescents in South Africa and in infants in The Gambia ^47^. Overall, M72/AS01E was found to be safe and well tolerated in the recruited populations, with more reactogenicity noticed in subjects with active TB disease. The vaccine induced a high-magnitude, M72-specific CD4+ T-cell polyfunctional response (IFN-gamma, IL-2, TNF-alpha and CD40L) that persisted for more than 1 year.

A phase 2b proof of concept efficacy study in approximately 3,500 IGRA positive, HIV-negative adults in clinics in South Africa, Kenya and Zambia, in which enrollees were followed up for three years for the occurrence of TB disease, has been concluded (NCT01755598). Subsequent to the meeting reported here, results have been released ^48^. The study demonstrated 54% (90%CI 14%–75%) vaccine efficacy against pulmonary tuberculosis, during a period of follow-up of approximately 2 years, the most promising results yet seen in clinical assessments of new TB vaccines. Increased reactogenicity (local reactions and flu-like symptoms) were more common in M72/AS01E vaccine recipients relative to placebo recipients, but no unexpected safety concerns were raised. Secondary analyses suggest vaccine efficacy does not appear to wane over the follow-up time, and may be a function of age, but secondary signals should be interpreted with caution, as they are based on small numbers. The study is ongoing, with final results expected to be released in 2019.

### c. H56:IC31

The H56:IC31 vaccine is an adjuvanted fusion protein, consisting of three highly immunogenic *Mtb* antigens – Ag85B, ESAT-6 and Rv2660c – and adjuvanted with the Valneva IC31© adjuvant ^49^. ESAT-6 is a premiere virulence-associated antigen highly expressed throughout all stages of infection while Rv2660c is a stress-induced antigen, strongly associated with latent TB infection. The IC31 adjuvant consists of ODN1a, a TLR9 ligand, and a stabilizing molecule that helps establish depot formation. The central immunologic strategy of this vaccine is to generate long-lived, Th1-type T-cell immunity. The vaccine is being developed by the Statens Serum Institute (Denmark), with multiple partners including Valneva, GmBH (Austria), Aeras, the South African TB Vaccine Initiative (SATVI), The European and Developing Countries Clinical Trials Partnership (EDCTP) and the Research Council of Norway.

H56:IC31 is being developed to prevent TB disease in adolescents, adults and the elderly, as well as in children and infants, to prevent recurrent TB, and to shorten drug treatment for active TB and serve as adjunctive immunotherapy to patients with active TB, including those with drug-resistant TB. The targeted efficacy is ≥50%, with a duration of protection of ≥10 years. The vaccine is administered via intramuscular injection (IM) in two doses.

Phase 1 and phase 2 studies of safety, immunogenicity and dose finding have been completed in IGRA+ and IGRA- adults, IGRA- adolescents, and in adults completing treatment for active TB. Thus far, the vaccine has demonstrated no safety concerns. Immunogenicity studies demonstrate a strong induction of ESAT-6 responses both in IGRA+ persons and in those completing treatment for active TB ^49^. Antibody responses also were noted in a proportion of vaccinees. Along the therapeutic track, an ongoing, open label phase 2a trial in Norway is assessing the safety and immunogenicity of H56:IC31 given three months into active TB treatment as adjunctive immunotherapy with and without additional COX2-inhibition (NCT02503839). A major phase 2 PoR trial of H56:IC31 is being planned and will enroll 900 persons being treated for pulmonary TB; 450 receiving vaccine and 450 administered a placebo (NCT03512249). The primary objective of this trial is to accelerate the development of H56:IC31 toward a possible phase 3 PoR trial and licensure for this indication. Capacity building and studies of mechanisms of reactogenicity, immunogenicity and efficacy also represent important components of this trial.

### d. ID93/GLA-SE

ID93 is a fusion of four *Mtb* antigens with diverse roles that are recognized by T-cells isolated from *Mtb*-exposed individuals and lack human sequence homology ^50^. The fused proteins in the ID93 vaccine include Rv1813, an antigen up-regulated under hypoxic conditions; Rv2608, the PPE protein, probably associated with the *Mtb* outer-membrane; and Rv3619 along with Rv3620, both included among the EsX protein family of secreted virulence factors. GLA-SE is a synthetic TLR-4 agonist adjuvant, formulated in a squalene oil in a water nano-emulsion. GLA-SE has been demonstrated to be safe in humans, with thousands of doses delivered, induces a TH1-biasing immunological response, and production is readily scalable.

ID93/GLA-SE is being developed for two indications: as an immunotherapeutic agent to improve the outcome of drug treatment for active TB, and as a prophylactic vaccine to prevent infection with TB. Proof of principle for the ability of ID93/GLA-SE to enhance TB treatment has been demonstrated in mice, guinea pigs and non-human primates, where immunotherapeutic vaccination with ID93/GLA-SE in combination with multidrug therapy was associated with a polyfunctional Th1 response, improved bacterial clearance and reduced pulmonary inflammation ^51^. Protection against progression to pulmonary TB in a guinea pig *Mtb* challenge model was observed for more than 1 year ^52^.

Two phase 1, and one phase 2a clinical trial of ID93/GLA-SE in healthy adults in the United States and South Africa have been completed. These trials, assessing safety, immunogenicity and dosing approaches, have included persons not vaccinated with BCG, BCG vaccinated individuals, and persons who are IGRA-, IGRA+ and those with active TB disease. Results from these studies demonstrated the induction of a broad, polyfunctional T-cell response, an increase in multi-functional antibodies, and robust CD4+ T-cell responses in IGRA+ adults, suggesting that the vaccine boosts the immune response to natural infection ^50^. The phase 2a trial, in which vaccination occurred at the end of TB treatment, demonstrated encouraging CD4+ T-cell and antibody responses to vaccination (NCT02465216). These studies also demonstrated an acceptable safety profile for ID93/GLA-SE in more than 200 research participants. Several new Phase 2 studies of ID93 / GLA-SE are being prepared. A Phase 2a study to evaluate the safety, immunogenicity and preliminary efficacy of ID93 / GLA-SE in preventing infection with TB among high-risk health care workers in Korea is being planned. Additionally, two Phase 2b clinical trials of ID93 / GLA-SE to evaluate the vaccine as an immunotherapeutic adjunct to multidrug therapy have received funding. In India, a collaboration with the All India Institute of Medical Sciences will administer the vaccine to patients undergoing multidrug therapy for both drug-sensitive and drug-resistant TB. In South Africa, plans are being developed for an expansion of the Phase 2a prevention of recurrence study, with the intention to advance administration of the vaccine proximally towards the initiation of TB therapy, beginning at the completion of therapy and moving as early as two months after chemotherapy has started.

### e. RUTI ™ for adjunctive immunotherapy

RUTI is being developed as an immunotherapeutic agent for adults, intended to improve the efficacy and shorten the duration of drug treatment for cases of active TB, including drug-resistant TB ^53^. RUTI is made of cell wall fragments of *Mtb* formulated in a liposome suspension and is administered subcutaneously (SQ) in a single dose. Preclinical experiments demonstrate an added effect compared to *Mtb* drug treatment in reducing *Mtb* colony counts in mice, a reduction in regrowth rate as far out as week 28 following *Mtb* challenge in guinea pigs, and a diminished TB relapse rate in a paucibacilli murine model of TB.

Two clinical trials have been completed. The initial study, a phase 1 safety, immunogenicity and dose-ranging trial in 24 healthy adults in Spain, triggered a vaccine-specific immunological response against several *Mtb* antigens, without significant toxicity, supporting further development ^54^. The second study was a phase 2 safety, immunogenicity and dose ranging trial in 48 HIV+ and 48 HIV- persons with LTBI in South Africa ^53^. These studies demonstrated a good cellular polyantigenic response after a first injection of 25 mcg in both HIV- and HIV+ participants and elicited a long-term memory surrogate response compatible with a prophylactic potential already observed in animal models.

Future development will focus on utilizing RUTI as an adjunct to drug treatment in patients with MDR-TB, to prevent disease relapse after treatment for drug-sensitive TB, and to shorten the drug treatment regimen. A phase 2a trial to evaluate the safety and immunogenicity of RUTI in MDR-TB patients favorably responding to standard MDR-TB treatment is scheduled to begin early 2019, with a plan for a subsequent phase 2b-phase 3 pivotal clinical trial in this population (NCT02711735).

### f. Vaccae™

The Vaccae™ vaccine is a heat-killed preparation of *Mycobacterium vaccae*, an NTM closely related to *M. obuense* ^55^ *.* Vaccae™ has been licensed in China as adjunctive immunotherapy for drug treatment of active TB. A pooled analysis of data from trials of the *M. vaccae* vaccine suggested some potential utility when used as an immunotherapeutic adjunct to drug treatment of active pulmonary TB ^56^. In 2012, the Chinese government approved the initiation of a clinical trial to test the candidate vaccine for prevention of the occurrence of tuberculosis in subjects with LTBI. A 10,000-person, placebo-controlled phase 3 study involving a 6-dose vaccination regimen was initiated (NCT01979900). Retention rates in the trial were high, and more than 100 cases of TB occurred during the study. The efficacy and safety data from this completed study have not yet been released.

## 4. Diversifying TB vaccine platforms

### a. Importance of immune characterization

Most advanced vaccine candidates share one important commonality: they were selected for development on the basis of their ability to induce interferon-gamma producing T-cells obtained from vaccinated animals and individuals upon *in vitro* mycobacterial antigen stimulation. Very little evidence, however, is available to confirm the full scope of the critical immune responses necessary to control TB disease and *Mtb* infection, including the potential roles of CD8+ T cell responses, tissue resident memory T-cells, mucosal-associated invariant T (MAIT)-cells in the lungs and B cell activation. Studying these and other diverse immune responses generated by current and future vaccines likely will be critical to advancing TB vaccine development in a more efficient and effective manner ^57^.

Many of the more diverse immune responses likely to be important in controlling TB in humans may not occur in small animal models, or even in non-human primates. Accordingly, “experimental medicine” studies, involving 20–30 people and designed to gain a better understanding of a vaccine’s ability to generate the types of immune responses that may be critical in controlling TB, particularly those occurring in lung parenchyma, in bronchus-associated lymphoid tissue, and in the pulmonary mucosa, will be important to conduct. It also remains important to engage in parallel, more empirical vaccine efficacy and safety studies designed to inform immune mechanisms of protection. Collection of biospecimens in clinical studies in support of planned or future immunological studies, using the best technology available, is critical to permit retrospective analyses of correlates of protection once clinical protection is demonstrated by a vaccine under development.

While it is important to develop and utilize a TB vaccine as soon as possible, it also is important to remember that evidence of TB disease has been discovered in humans occurring as far back as 9000 BCE ^58^. Given this lengthy history between TB and humans, it is critical that we take the long view when developing vaccines for TB; whether we develop a vaccine by 2035 or by 2050 represents a rounding error from the perspective of human history.

### b. Cytomegalovirus (CMV) recombinant vaccines against TB

*Mtb* represents a particularly difficult challenge for vaccine developers as it appears to both evade host immunity and modulate it in its favor. Accordingly, a successful TB vaccine likely will have to hit *Mtb* infection hard and early, in a manner that prevents *Mtb* from taking control of the host immune response directed against it. A CMV-vectored vaccine strategy offers the potential for accomplishing this by generating “effector-memory” T-cells prepositioned in the lungs, at sites of early pathogen colonization ^59^.

The strategic rationale for selecting a CMV vector for a TB vaccine resides in its unique ability to elicit and maintain robust, effector-differentiated CD4+ and CD8+ T-cell responses in organs outside lymphoid structures, including the respiratory tract. Experiments with rhesus CMV (RhCMV) in rhesus macaques (RMs) demonstrate that RhCMV vectors expressing foreign antigens manifest the critical biological properties needed to generate effector-memory T-cells in a safe manner. These properties include the ability to super-infect rhesus already infected with CMV, and to persist indefinitely despite robust anti-CMV immunity in naturally CMV seropositive animals; the capacity to elicit and indefinitely maintain high frequency, “effector differentiated” T-cell (T effector and T tissue-resident effector) responses in mucosal sites, lymphoid tissues and parenchymal organs; and the ability to maintain immunogenicity despite profound attenuation. In particular, genetically attenuated (ULb’ mutated) RhCMV vectors (68-1 RhCMV) demonstrate an unexpected and unusual capability to elicit CD8+ T-cell responses that entirely, and broadly, target novel epitopes restricted by MHC-E and MHC-II ^60^.

The primary rationale for a CMV-vectored TB vaccine is to generate tissue effector T-cells capable of immediate interception of *Mtb*-infected macrophages at sites of initial lung infection prior to the initiation of the substantial immune system modulation caused by *Mtb* necessary to permit *Mtb* survival and replication. Two RhCMV-TB vaccines have been created thus far, one expressing nine *Mtb* proteins across four different RhCMV vectors, the other expressing six *Mtb* proteins as a single polyprotein from one vector. Two RhCMV-TB efficacy pre-clinical assessments studies of these vectors, utilizing a low-dose (25 colony forming unit (CFU)) challenge of Erdman strain *Mtb*, have been completed ^59^. In the first study, the extent of lung, non-lung and overall TB disease at one year following *Mtb* challenge was significantly reduced in RhCMV-TB vaccinated NHPs as compared to unvaccinated and BCG vaccinated controls. BCG vaccination prior to RhCMV-TB vaccination appeared to reduce the efficacy of the experimental vaccine. In the second study, no TB disease was detected via lung CT scans in 13 of the 27 CMV-TB vaccinated animals, as compared to unvaccinated controls where all demonstrated TB involvement of lungs and lung-draining lymph nodes. While all unvaccinated RMs demonstrated both pulmonary and extra-pulmonary TB at necropsy, no gross or microscopic TB was found in the 13 RMs with CT scans that did not demonstrate TB disease. Moreover, 10 of these 13 were *Mtb* culture negative. Taken together, these studies demonstrated unequivocal protection—defined as a finding of no granulomatous disease at necropsy (41%) and a significant reduction in TB disease (32%)—in 73% of vaccinated RMs. Comparisons between different CMV-TB constructs also determined that efficacy is not dependent on unconventional MHC-II and MHC-E restricted CD8+ T-cell responses.

Future steps in the development of these promising vaccine constructs will include optimization of the vector backbone and the gene inserts and validation of the final CMV-TB vector design in NHP challenge experiments. The first phase 1 clinical trial of a CMV-TB vaccine is targeted for 2020.

### c. Novel emerging vaccine technologies: mRNA-based vaccines

Messenger RNA (mRNA) vaccines represent a non-viral delivery system of vaccine antigens that offers the potential of stimulating both the innate and adaptive immune systems in a manner that provides a balanced cellular and humoral antigen-specific response ^61^. Factors spurring interest in mRNA vaccines include their ability to express complex membrane proteins, induce both CD4+ and CD8+ T-cell responses (unlike many protein antigens), permit efficient re-dosing given the absence of anti-vector immunity and their non-cell based manufacturing platform, allowing more rapid production than for vaccines requiring cell culture-based manufacturing processes. Potential cons to utilizing mRNA vaccines include the reactogenicity of the delivery systems currently in use which limits the mRNA dose and antigen multiplicity, and cost-of-goods considerations given target price points for a TB vaccine.

No mRNA vaccine as yet has been developed for TB. mRNA technology, however, is a potentially attractive vaccine strategy against TB in light of the platform’s ability to induce potent T- and B-cell responses, including polyfunctional T-cell responses that home to the lungs, the ability of these vaccines to co-express multiple antigens, and the potential to deliver these vaccines via ID, IM, subcutaneous (SC), intranasal (IN), aerosol (AE) and intravenous (IV) routes, based on the formulations. Limitations to their use for TB include the non-persistent nature of antigen expression and the limited number of antigens that can be included in each vaccine. Ultimately, the identification of a set of *Mtb* antigens that offer the possibility of effective protection would be needed to stimulate greater interest in applying mRNA vaccine technology to TB prevention efforts.

### d. New candidates for live attenuated TB vaccines

Live, attenuated vaccines (LAVs) are pathogens missing genes responsible for producing virulence factors or key metabolic enzymes, with the absence of these genes resulting either from rational design or randomly through passage. When creating LAVs as vaccine candidates, developers must maintain a balance between attenuation and virulence, as too much attenuation may impair the ability of the candidate to generate a sufficiently protective immune response, while insufficient attenuation would raise safety concerns.

Major advantages to using LAVs include their ease and low cost of production, their longer persistence and the ability to stimulate both adaptive and innate immune responses, thereby obviating the need for adjuvants with the establishment of a long-term, comprehensive immunity. This stands in contrast to inactivated whole cell vaccines that primarily induce neutralizing (humoral, antibody-based) immunity with minimal mucosal and innate immune stimulation and little generation of cell-mediated immunity (CMI); and recombinant vector vaccines that primarily generate CMI with minimal innate and mucosal immunity, and with little to no humoral immunity ^62^.

The primary disadvantage to LAVs is a concern about their safety. The potential for reversion to wild-type virulence remains a possibility for organisms attenuated through the deletion of one or a few virulence genes. Additionally, LAVs that behave in an attenuated fashion in persons with normally functioning immune systems may prove dangerous and even deadly if administered to persons with compromised immune function.

Rational deletion of *Mtb* virulence genes represents an exciting approach to creating a new generation of TB vaccines. One attenuated *Mtb* vaccine concept results from the deletion of MosR (Regulator of Mycobacterial Operons of Survival, Rv0348) that plays an important role in *Mtb* survival during infection ^63^. C57BL/6 mice vaccinated subcutaneously with the ΔMosR vaccine demonstrated a low level of vaccine persistence in lung and spleen for at least 16 weeks following vaccination. After challenge with the highly virulent *Mtb Beijing* strain, improved protection over BCG-vaccinated mice was observed; in some animals *Mtb* could not be detected at 60 days after challenge, with benign-appearing lungs on histopathological examination ^64^.

In order to reduce the chance of reversion to a virulent, wild-type state, another gene deletion, involving the echA7 gene, has been introduced. A plan is underway to combine these two deletions into the same *Mtb* strain and assess the potential utility of this construct as a TB vaccine.

### e. A systematic antigen discovery approach in humans

The current approach to TB vaccine development suffers from three shortcomings: 1) it has focused mostly on a narrow set of candidate TB antigens which may have suboptimal activity in protecting against TB; 2) it has focused on generating classical, CD4+ T(h1) cells, which may be essential but not sufficient to generate an optimally protective response; and 3) has not taken into account recently emerging evidence suggesting a role for traditionally “ignored” cells, such as B-cells, in generating immunological protection against TB. Accordingly, exploring the role of unconventional T-cells and T-cell responses, such as donor unrestricted T-cells (DURTS), mostly CD8+ T-cells restricted by CD1, MR1, HLA-E, TCR-gamma-delta; non-IFN-γ producing T-cells; and the role of non-T-cells will be critical to future TB vaccine development efforts ^57, 65^.

The current global clinical pipeline of TB vaccine candidates utilizes a limited number of the approximately 4,000 *Mtb* antigens. A critical question is whether the optimal antigens are being selected for inclusion in protein subunit and recombinant viral vaccines. In a large phase 2b trial of the MVA85A vaccine in South African infants, the vaccine did not provide significant additional protection above BCG vaccination ^66^. A possible explanation for this outcome is that Ag85A, although highly immunogenic, may not have been the right antigen given that its expression is downregulated later in infection and may not be presented sufficiently by infected cells.

An emerging hypothesis in defining optimal antigens to include in a TB vaccine is to identify *Mtb* antigens expressed during infection in the lungs of susceptible individuals ^67– 69^. To identify such antigens, an unbiased, genome-wide antigen discovery approach was taken, utilizing *Mtb* RNA isolated from the lungs of four different mouse strains, ranging from hyper-susceptible to TB (C3H/FeJ mice, the sst1 strain) to genetically resistant (C57Bl6 mice). Utilizing a genome wide qRT-PCR platform developed at Stanford University, *Mtb* genes were selected that were persistently and highly expressed *in vivo* ( *in vivo* expressed – IVE-TB – genes) at multiple time points following AE *Mtb* infection. 194 IVE-TB highly expressed genes were identified and 50 further selected based on ranking in the top 15% during infection; hyperconservation with wide HLA coverage and/or homology with *Mycobacterium leprae.* Many of these IVE-TB antigens were found to induce strong CD4+ T central memory and CD8+ T-cell responses in PBMCs from long-term, latently *Mtb* infected individuals, and were recognized by both T- and B- cells ^69^.

Almost all *Mtb* antigen discovery approaches have mainly relied on IFN-γ measurements. Recent evidence, however, suggests that IFN-γ only contributes marginally to overall protection against TB in the lungs of mice ^70^. An investigation of other responses, including proliferative and cytokine profiles besides IFN-γ, in a cohort of healthy Dutch individuals with PPD and/or ESAT6+CFP10 *in vitro* responses, and in a cohort of Norwegian individuals with LTBI, demonstrated that many IVE-TB antigens induce cytokines other than IFN-γ ^71^.

These new approaches may lead to novel classes of antigens with promising vaccine potential. Additionally, B-cells play a critical, underappreciated role in conferring immunity to human TB ^72, 73^. B-cells may provide an important, novel target for TB vaccination, but *Mtb* antigens that activate B-cells have not been extensively studied as yet.

### f. Antibody-generating vaccines for TB

It has long been appreciated that T-cell responses are critical in controlling TB. T-cells do more than direct CMI responses, however, as they also provide a crucial link to humoral immunity based on B-cell responses.

Experiments in mouse models of *Mtb* infection demonstrate that depleting B-cells decreases mouse survival following *Mtb* exposure, while B-cell depletion followed by B-cell restoration returns survival to the degree observed in control mice, suggesting an important role for B-cell responses in controlling TB ^73, 74^. Moreover, histopathological studies in *Mtb*-infected humans and NHPs demonstrate aggregates of B-cells proximal to pulmonary granulomas, with B-cell laden tertiary germinal centers surrounding the granulomas ^75, 76^. Antibody-deficient mice also demonstrate a greater degree of viable *Mtb* in both lungs and spleen, and a marked decrease survival after infection with *Mtb* ^77^. Passive transfer experiments with the monoclonal antibodies anti-lipoarabinomannan (LAM) and anti-HspX (directed against the 16 kD protein on *Mtb* outer membranes expressed during times of mycobacterial stress, including within granulomas) resulted in improved survival (with anti-LAM antibodies) ^78^ and decreased lung *Mtb* CFUs (with HspX antibodies) ^79^ in mice following *Mtb* challenge. These experiments provide additional evidence that antibodies limit *Mtb* infection. Improved mouse survival also was seen following administration of monoclonal antibodies directed against the mycobacterial heparin-binding hemagglutinin (HBHA), with no effect on diminishing lung *Mtb* CFUs but with a marked decrease in spleen *Mtb* CFUs, suggesting an effect on decreasing *Mtb* dissemination ^80^. Additionally, the particular isotype of antibody makes a major difference in the ability to control *Mtb*, with IgA monoclonal antibodies having a larger effect than IgG1 antibodies ^81^.

Most clinical studies of TB vaccines have not studied the role that antibodies may play in contributing to prevention of TB. Accordingly, little thought has gone into defining vaccine-induced antibody target product profiles (TPPs). In creating an antibody TPP for TB vaccines, it is important to recall that antibodies do much more than recognizing and blocking infection via pathogen neutralization. By binding to antigens on the surface of infected cells, antibodies play crucial role in stimulating a broader and more effective immune response, including the inducement of cytokine secretion to recruit cells such as dendritic cells and T-cells involved in mediating CMI, inducing autophagy in infected cells, and prompting innate immune responses including the recruitment of neutrophils, natural killer (NK) cells, monocytes and phagocytes.

Advances in systems serology assays now permit qualified and validated approaches to assessing antibody qualities and functions. Studies of TB patients utilizing systems serology have discovered that qualitatively different TB-specific antibodies are induced and distinct innate immune recruiting profiles are manifest in persons with LTBI as compared to those with active TB disease ^82^. Antibodies play a key role in creating these differences, as antibodies expressed in persons with latent infection help stimulate innate immune responses by attracting dendritic cells to macrophages infected with *Mtb*, resulting in dendritic cell-mediated activation of innate immune responses. In particular, antibodies expressed in LTBI prepare the immune system to kill *Mtb*-infected cells, a signal sent via glycosylation patterns on the Fc domain of antibody molecules that were naturally modified during *Mtb* infection to access different innate immune functions ^83^. Identifying the Fc glycosylation patterns that result in enhanced innate immune responses to TB provide a potential target for vaccine developers attempting to selectively induce such antibodies via vaccination.

Antibodies also play a unique antimycobacteriological role by identifying cells infected with *Mtb* and restricting *Mtb* survival. Non-classical killing mechanisms triggered by antibodies include enhancement of opsinophagocytosis, lysosomal maturation, and inflammasome activation via a metabolic rewiring of macrophages, resulting in enhanced restriction of *Mtb* growth.

Clinical trials of vaccine candidates provide important opportunities to profile the quality of antibodies generated by these vaccines. Additionally, utilizing adjuvants offers an opportunity to generate many distinct antibody responses, resulting in the potential of rationally choosing adjuvants to more specifically stimulate the types of antibody responses that may be optimal in contributing to the control of TB. Accordingly, it will be critical to continue the clinical assessment of TB candidates and to build into these trials the opportunity to assess antibody responses as well as CMI.

Four hypotheses have emerged to explain the possible role of antibodies in contributing to the control of *Mtb* infection and TB disease. The first hypothesis involves restricting the extent of initial *Mtb* infection in and around the alveolus by blocking *Mtb* physically, by inducing complement-mediated *Mtb* killing, and by stimulating enhanced phagocytic activity against *Mtb*. The second proposed mechanism involves balancing inflammation resulting from *Mtb* infection, mainly by redirecting pathologic innate immune activity in a way that reduces corresponding immunopathology. The third proposed mechanism involves regulation of granulomas, including prevention of granuloma formation (in light of the emerging theory that granulomas are used by *Mtb* to more efficiently spread infection via attraction of susceptible macrophages) and the clearance of *Mtb*-infected cells. Finally, antibodies may enhance dendritic cell and T-cell antigen presentation and regulation, through signals sent by specific Fc glycosylation patterns.

Studies of antibody responses in LTBI populations, individuals who appear to be resistant to *Mtb* infection despite ongoing exposure, and persons participating in TB vaccine trials or in experimental medicine studies, utilizing more aggressive serological sampling techniques such as plasmapheresis, will be critical to advance the understanding of the potential roles played by antibodies in controlling TB. Using this information to design vaccine strategies that stimulate both humoral and CMI responses effective in controlling TB represents an important new strategy in TB vaccine development.

### g. Diverse T-cell responses against *Mtb*

An important characteristic of a vaccine-stimulated immune response to *Mtb* is the ability of T-cells to recognize an *Mtb*-infected cell via receptors that are highly conserved in humans. Most vaccines currently being developed do this through “classical” HLA-I or HLA-II restricted antigen presentation. Donor unrestricted T-cells (DURTS) represent different types of T-cells that interact with human antigen-presenting cells (APCs), including dendritic cells or macrophages, through mechanisms other than classical HLA-I or HLA-II restricted antigen presentation. Examples of restriction molecule ligands associated with DURTS include: CD1a, b, c molecules, which present lipid and glycolipid moieties and bind CD1-restricted T-cells, including Type I and Type II natural killer T-cells (NKT cells); MR1, which presents riboflavin metabolites and interacts with mucosal-associated invariant T-cells (MAITS); and HLA-E, a highly conserved receptor that recognizes a diverse array of peptide ligands, binds to HLA-E-restricted T-cells and helps to mediate natural killer-cell killing ^84^. Although our understanding of DURTS ligands is still relatively superficial given that this is a young field of investigation, the potential applicability to TB vaccine development is great given that virtually all of the types of antigens presented by these ligands can be found in *Mtb*.

Studies of DURTS suggest that they contribute to protective immunity, with MR1 restriction involved in responses against *Mycobacterium bovis* via BCG vaccination ^85^, and *Klebsiella pneumoniae* ^86, 87^, and both CD1 ^88^ and HLA-E ^89, 90^ mediated responses contributing to *Mtb* control. It is not yet clear, however, if these are memory responses that can be generated through vaccination. Additionally, the duration of such responses is not known.

MR1-restricted responses are highly conserved in mammals and do not diverge across mammal species. MR-1 interacts with MAITS as sensors of the metabolome via riboflavin metabolites, but also through other, as-yet-unidentified molecules ^91^. In studies of patients with TB, a dramatic enrichment of MAITS was found in samples obtained from bronchoscopies as compared to peripheral blood, with selective enrichment of certain T-cell receptors (TCRs) identified in the bronchoscopically-obtained samples, suggesting antigenic discrimination. This finding led to the discovery of a novel class of MR1 ligands called photolumazines. Experiments with synthetic photolumazines suggest that there is immunological memory underlying MR1-mediated antigenic recognition that could be harnessed in a TB vaccine ^92^.

CD1-restricted T-cells recognize glycolipid antigens ^93, 94^. They represent an intriguing area of study given the presence of known antigenic glycolipids in *Mtb*, although the absence of group 1 CD1 molecules in mice increases the difficulty of assessing the importance of CD1-restricted immune responses in protecting against *Mtb*. Additionally, it is not yet known whether immunologic memory can be imparted to CD1-restricted T-cells.

HLA-E restriction encompasses presentation of both peptidic and non-peptidic antigens, and initially was identified as an inhibitor of recognition between NK cells and T-cells. Subsequently, however, HLA-E-restricted T-cell clones have been found to manifest antimicrobial properties, including the inhibition of intracellular *Mtb* growth ^95^. This inhibition appears to be mediated both by generating cytolytic properties in the T-cells, and by inducing a cytokine profile from these T-cells that resemble a Th2 phenotype. Additionally, gamma-delta cells, specifically γ9δ2 cells, have been found to be expanded following vaccination with BCG ^96^ with further investigations suggesting a role in controlling intracellular growth of *Mtb* ^97^.

Further elucidation of the role of DURT-mediated immune control of *Mtb* will most likely depend on the development of fit-for-purpose animal models, particularly mouse models, engineered to assure the relevance of DURT response assessments. An example of this approach is the development of a humanized transgenic mouse model which expresses group 1 CD1 ^98^.

A critical question regarding the potential for targeting DURTs through vaccination is whether DURTs have memory. If they do, and if durable expansion and preferential recall responses to these novel ligands can be elicited through vaccination, then DURTs could be harnessed directly as a potentially powerful vaccination strategy. If they are not capable of developing an anamnestic response but, instead, function more as adjuvants, then DURTs could be manipulated to help facilitate the acquisition of adaptive immune responses to *Mtb*. Ultimately, it will be necessary to develop fit-for-purpose animal models, identify the appropriate immunogens, develop techniques to formulate the ligands and create relevant animal challenge models to explore the potential of harnessing DURTs as a vaccination strategy against TB.

### h. Immune correlates and signatures of protection

Biomarkers that can reliably and accurately predict clinical outcomes of diseases are invaluable to vaccine development efforts. Biosignatures, custom-made combinations of biomarkers, offer the potential to enhance the predictive power of a single biomarker. Principally, biosignatures can be useful both in selecting promising vaccine candidates for more advanced development, while rejecting those with a reduced probability of ultimate success. They also can be used to more finely tailor the selection of individuals being enrolled into clinical trials, thereby reducing the cost and increasing the efficiency of such trials. Thus far, studies exploring blood, urine or breath of persons with asymptomatic *Mtb* infection have not identified a biosignature predictive of the development of active TB disease.

The development of advanced, data-dense assessment techniques, including transcriptomics, proteomics, metabolomics has enhanced the opportunity to identify biosignatures of the progression of *Mtb* infection to active TB disease. Transcriptomes, which require samples of whole blood, combined with computational modelling, currently represent the most widely studied biomarkers in TB. Taken together, small signatures, utilizing three to four transcripts and assessed via decision trees or other computational methods, have resulted in a biosignature which discriminates active TB disease from LTBI with 85–90% sensitivity and specificity ^99, 100^. Signatures comprising 16 transcripts have been shown to be associated with the future development of active TB in individuals with LTBI 6–12 months prior to a clinical diagnosis ^101^. Further refinement of algorithms led to a predictive signature composed of only two transcript pairs which detect risk of active TB up to one year prior to clinical diagnosis of TB in different regions of the African continent ^102^. Metabolomics represents an under-researched area, particularly given that such studies can be run utilizing serum or plasma rather than whole blood, reducing the complexity and cost of sample collection and storage. A prospective multisite study across sub-Saharan Africa analyzed metabolic profiles in serum and plasma from HIV-negative, TB-exposed individuals who either progressed to TB 3–24 months post-exposure (progressors) or remained healthy (controls) ^103^. A metabolic biosignature of TB within months of diagnosis, with 69% sensitivity at 75% specificity, was found. These biosignatures are consistent with a state of subclinical disease prior to manifestation of active TB, potentially enabling interventions to prevent disease progression and transmission.

While these findings have relevance to decision-making around initiating preventive drug therapy before the development of clinical disease, they also provide the opportunity to stratify individuals in clinical trials of vaccines. Using these techniques to identify and selectively enroll LTBI individuals who are at risk of developing TB in an efficacy trial of a vaccine to prevent TB disease could considerably reduce the numbers of study participants required, shorten the clinical trial duration and greatly reduce cost by several orders of magnitude, assuming that these signatures could be utilized at reasonable cost and speed.

Important future directions in biosignature research relevant to TB vaccine development will be to identify signatures of vaccine efficacy and signatures of vaccine safety. While studies of BCG responses can lay the groundwork for this ^104, 105^, ultimately this effort will depend upon identifying an initial, partially efficacious TB vaccine through ongoing clinical trials, and having had the foresight to bank sufficient blood and serum specimens from trial participants to permit biomarker studies ^106^. This further emphasizes the need to continue to conduct clinical efficacy trials of TB vaccines, and to invest in the collection of biological specimens from the participants. Only through efforts such as this will unexpected findings be identified that potentially could result in a major acceleration of novel TB vaccine development.

## 5. Use of models in translational TB vaccine research

### a. Small animal models

Effective natural immunity against developing TB exists, given that 90–95% of *Mtb*-infected individuals fail to develop active TB disease ^1^. While this rate of natural immune protection is difficult to improve upon, it will be necessary to do so to control the global TB epidemic. In order to effectively manipulate the human immune response to TB we require new and diverse vaccine candidates and we need to correlate the immune responses induced by these vaccines with the degree of protection conveyed. Small animal models of *Mtb* infection can assist in this effort ^107^.

Small animals can be experimentally manipulated to provide a reproducible challenge model capable of screening vaccine modulated vertebrate immune responses to limit *Mtb* growth in the lung. Mice and guinea pigs (GPs) have been the main small animal species utilized for assessing vaccine candidates ^107^. Mice are inbred, easily manipulated, inexpensive, and there are extensive reagents for immunological studies. Both mice and GPs provide the opportunity to compare vaccine efficacy by assessing bacterial load and survival following *Mtb* challenge. GPs are susceptible to very low dose *Mtb* challenge and can be used for natural transmission or repeated low- and ultra-low-exposure models. It may be feasible to utilize GPs in a post-exposure model as well. The pulmonary pathology of *Mtb* infection in GPs is similar to that of human primary TB ^108^.

Potential indications for TB vaccines in humans include prevention of TB disease (PoD), prevention of relapse or reinfection following treatment (PoR), and prevention of *Mtb* infection (PoI). To discriminate between potential PoD vaccines, TB challenge in vaccinated small animals provides a direct measure of an anti-mycobacterial host response as well as the opportunity to detect pathological outcomes. For meaningful PoD efficacy, the optimal outcome in a small animal would be to reduce bacterial burden below the level of detection. Assessment of PoR vaccines can also be performed in small animals, but due to the variability in outcomes, large numbers of mice are required ^109^. A PoI vaccine model is not currently optimized for small animals, although studies of GPs in a natural exposure environment could provide proof of concept ^17^.

Other factors, such as the nutritional status of the animals, corticosteroid stress, the animal microbiome and the genetic background of the animal can influence the outcomes of challenge experiments. The development of “collaborative cross” (CC) mice, a multiparent panel of recombinant inbred mouse strains derived from eight founder laboratory strains ^110^ provides a striking demonstration of the effect of genetics on response to vaccination and *Mtb* challenge, as shown by the differential effect of BCG vaccination on the genetically diverse CC mouse strains ^111^. Additional factors that can affect outcomes are the bacterial strain selected for the challenge ^112^ and the bacterial dose. While these external factors should be controlled for during vaccine screening, they also provide the potential for testing vaccine efficacy under a variety of conditions.

The revised TBVI/Aeras stage gate criteria make experiments in small animals an explicit part of TB vaccine candidate development ^113^. Stage gates B and C include calls for exploration of safety, immunogenicity and protection in small animal species. In this context, protection is defined as being reproducibly and statistically better at preventing TB disease than BCG or a relevant benchmark. Ultimately, it will be important to determine the extent to which vaccine protection demonstrated in small animal *Mtb* challenge experiments is corroborated by data from experiments in NHPs and in human clinical trials.

Assessment of TB vaccine candidates in small animal models can be compromised by suboptimal experimentation as well as inappropriate or over-interpretation of experimental outcomes. It is important therefore to state that there are no ‘bad’ models, only inappropriate interpretation of the results.

### b. The role of a continuous exposure natural transmission animal model for vaccine development

Standard animal challenge models of *Mtb* infection differ from natural infection in a number of ways, including challenge with a higher *Mtb* exposure than the very low exposures that characterize natural infection; a single challenge as compared to the repeated exposures associated with natural infection; and exposure to a naturally occurring form of *Mtb* rather than laboratory-grown strains. Laboratory-grown strains are usually treated with mild surfactants such as Tween™ that prevent clumping and permit accurate quantification of dose, but strip the outer lipid capsule off of *Mtb* and thereby potentially alter its immunogenicity ^114^. To overcome these issues, a human to guinea pig (GP) continuous natural exposure model was established ^17^. In this model ambient air from a specially built patient unit caring for individuals with active TB is removed via negative pressure and exhausted into a neighboring facility harboring GPs, with each GP receiving an average 4-month exposure to the stream of air laden with naturally occurring, uncultured *Mtb*. A total of 362 GPs have been exposed over 12 years: 91 (25%) remained free of infection while 271 (75%) became infected as determined by tuberculin skin test (TST) conversion. Of the 271 TST converters, 53 (15%) experienced a reversion of their skin test, with 33 (9%) of the reverters subsequently reconverting, presumably due to re-infection. Only 54 (15%) of the infected or re-infected GPs went on to develop TB disease.

TST reversions following initial conversion occurred in 15% of convertors, more commonly than had been anticipated. Repeat TST conversion following initial reversion was completely eliminated by irradiating the air from the patient unit, suggesting that the reversion and repeat conversion events were due to subsequent re-infection rather than non-specific reactions to non-viable *Mtb*. Examining the time course of TST reactions in these GPs over time revealed a pattern suggesting repeated *Mtb* exposures and subclinical infections, many which initially resolve but which ultimately result in established infection and disease.

These findings illustrate the critical role that repeated reinfection with *Mtb* appears to play in the overall course of TB pathogenesis. A correlative finding was described in a study of TB patients in a Siberian hospital, where admission into the hospital with drug-sensitive TB (DS-TB) translated into a six-fold increased risk of developing drug-resistant TB (DR-TB), as compared to patients with DS-TB treated as outpatients, due to re-infection with DR-TB while in hospital ^115^.

Preventing disease due to re-infection with new *Mtb* strains represents an important target for TB vaccines but a difficult one to assess given the absence of a biomarker for *Mtb* reinfection. A continuous exposure, natural transmission animal model that mimics human exposure in many high-burden settings, such as the human to GP exposure model described here, can serve to address this need. Vaccine suppression of transient TST or IGRA conversions seen in this model may represent an important target. A clinical study also is being implemented to assess the role that BCG vaccination may play in preventing IGRA-negative, BCG-naïve individuals who will be working in TB-endemic areas from acquiring *Mtb* infection. This investigation, called the Tuberculosis Immunization to Prevent Infection (TIPI) study, will enroll 2,000 USA-based healthcare and humanitarian workers (e.g., Peace Corps workers, persons entering service with Doctors without Borders or other NGOs). Half will be will vaccinated with BCG, and IGRA conversion rates will be compared against the unvaccinated arm over the period of exposure. Blood samples also will be collected to permit an assessment for correlates of immune protection if a difference in IGRA conversion rates is found between vaccinated and unvaccinated cohorts.

### c. Simian models of TB

Non-human primates (NHPs) represent the closest model of human *Mtb* infection ^116^. NHPs demonstrate the full spectrum of TB, including active disease, latent infection and reactivation ^117, 118^. TB-induced pathology in NHPs is reflective of human pathology, including caseating granulomas and other granuloma types, as well as cavitary lung disease ^118^. Additionally, NHPs are immunologically closer to humans than other animal models as they possess unconventional T-cell subsets, including CD1a-d and delayed type hypersensitivity (DTH) responses to BCG and TB ^116^. Reagents and laboratory technologies are available for the detailed analysis of the immune system of macaques, a NHP species frequently used to study TB pathogenesis and in *Mtb* challenge experiments. Sequential sample collections from blood and mucosal sites also are possible, permitting time course studies of systemic and mucosal immunity that enable studies of biomarkers and immune correlates of protection.

Rhesus macaques, mainly of Indian origin, and cynomolgous macaques currently are the most common NHP species used to model *Mtb* infection and TB disease ^119^. One major advance in improving NHP models of *Mtb* challenge applicable to vaccine candidate assessment has been the development and application of advanced, non-invasive imaging techniques to serially track disease progression in a sensitive and quantifiable manner. The simultaneous application of computerized tomography (CT), which provides detailed images of internal structures and permits the quantification of pulmonary disease burden, in combination with positron electron tomography (PET), which demonstrates the spatial distribution of metabolic activity, thereby providing fundamental information relevant to TB pathogenesis, have proven valuable in assessing the degree of protection provided by newly-developed vaccine candidates ^20^.

Another major improvement in NHP models of TB has been the development of low-dose *Mtb* challenge (<25 CFU) and very-low-dose *Mtb* challenge (<10 CFU), doses that more closely approximate natural human exposure to *Mtb* than did the 500–3,000 CFU exposures previously used. Additionally, the development of advanced tools for studying immune responses, permitting detailed studies of adaptive immune responses as well as assessments of diversified components of the rhesus immune response, have increased our understanding of NHP immune responses to vaccination and its effect on controlling an *Mtb* challenge before the candidate under study is selected for clinical evaluation.

As macaque populations demonstrate different responses to *Mtb* infection, it is important to select the rhesus species most relevant to the target product profile of the candidate vaccine when designing challenge experiments. It is also important to consider the species in which the experiments have been done when attempting to draw comparisons between challenge studies. For example, cynomolgus macaques demonstrate a greater degree of disease control following *Mtb* challenge than do rhesus macaques, making the former potentially more representative of *Mtb* infection in adults, and the latter modeling *Mtb* infection in infants and young children, where *de novo* infection tends to have more serious consequences ^120^. The ability to control *Mtb* infection also varies within species; among cynomolgus macaques, the Asian sub-species controls *Mtb* infection better than those of the Mauritian sub-species. Among rhesus macaques, Chinese rhesus control *Mtb* better than do those of the Indian subspecies. To further facilitate inter-study comparisons, a greater degree of harmonization and standardization of experimental methods must be achieved, including challenge strain selection, use of imaging, scoring gross pathology specimens and identifying priorities as to future experimental directions, such as assessing AE delivery of vaccines. This harmonization and standardization effort is underway within the Collaboration for TB Vaccine Development (CTVD), led by the Bill and Melinda Gates Foundation (BMGF). CTVD recommendations regarding NHP-based study design for TB vaccine development have recently been published ^121^.

### d. Achieving sterilizing immunity in animal challenge models

Most TB vaccine candidates reduce *Mtb* CFU by ~1–1.5 logs in mice challenge experiments and are usually considered sufficiently protective to merit further development if they result in a ~1 log improvement over the reduction seen by BCG. As even this degree of CFU reduction results in ~1×10 ^4^ *Mtb* CFU in mouse lungs following a standard *Mtb* challenge experiment, the question is whether this is a sufficient degree of CFU reduction to suggest the potential success of the vaccine candidate. The acceptance of this degree of post-vaccination pulmonary *Mtb* CFU has been largely based on the idea that this may be the full extent of suppression that TB vaccines can produce in mice, given the “limited dynamic range” of the murine immune system. It is unclear, however, whether this assumption is correct, or whether sterilizing immunity, or something approximating this, can, in fact, be generated in mice through manipulations of murine immunity that might be achievable through vaccination.

The standard mouse model of vaccination and *Mtb* challenge calls for vaccination at time 0, permitting 30 days to pass to allow the generation of a memory response, and then administering an AE *Mtb* challenge. Subsequently, the degree of protection afforded by the vaccination, and the *Mtb*-specific T-cell responses are assessed.

Challenge studies in vaccine-naïve mice reveal that the pulmonary accumulation of *Mtb*-specific T-cells during primary *Mtb* infection is delayed by approximately 21 days, with peri-granulomar B-cell follicles – inducible bronchus-associated lymphoid tissue (iBALT) – appearing at 24 days ^122^. Vaccinating the mice with BCG accelerates the *Mtb*-specific T-cell response by one week, with these cells appearing in the pulmonary parenchyma between 12–14 days following *Mtb* challenge and with iBALT appearing at day 17. In either case, *Mtb* is capable of establishing viable and progressive pulmonary infection, albeit with fewer organisms following BCG vaccination.

It is possible that the delay in appearance of *Mtb*-specific T-cell responses in the lungs may represent a critical bottleneck to developing effective TB vaccine-induced immunity, a bottleneck that could be overcome if *Mtb*-specific T-cells could arrive at the initial sites of infection earlier. To test this hypothesis, dendritic cells (DCs) pulsed with *Mtb* antigens were transferred intra-tracheally one day before and then four days after AE *Mtb* challenge to mice that had received BCG vaccination, and some of which had also received a mucosal boost with Ag85B administered intranasally. This DC transfer overcame the TB vaccine bottleneck, resulting in the presence of *Mtb*-specific, cytokine-producing T-cells on the third day following challenge, inducing iBALT 8 days following *Mtb* infection, and resulting in rapid lung macrophage induction in the vaccinated mice ^123^. The rapid vaccine-induced T-cell responses limited early *Mtb* growth, reducing *Mtb* CFU by 2 logs or more over vaccinated animals that had not received the DC transfer, with *Mtb* undetectable in approximately 50% of the mice upon sacrifice on day 20.

The DC transfer proved to be more effective in inducing sterilizing or near-sterilizing immunity in mice that had received the mucosal Ag85A vaccine in addition to BCG, as compared to mice that only had received BCG. Gene expressions studies revealed that the gene expression signature of T-cells induced by the vaccines and antigen-pulsed DCs were enriched for the mucosal/airway phenotype. When the experiment was repeated using a multi-drug resistant *Mtb* strain as the challenge strain, however, sterilizing immunity was not achieved. Additionally, endogenous, host DCs could be induced to generate near-sterilizing immunity through the administration of a select activation of innate mechanisms including CD103 and CD40 pathways. Delivery of amph-CpG adjuvant and CD40 agonist at the time of challenge, rather than through the transfer of pre-stimulated DCs also provided complete *Mtb* control, providing additional support for the possibility of stimulating this type of response through vaccination ^123^.

Hints at the possibility of achieving near-sterilizing immunity against *Mtb* through vaccination have occurred with *Mtb* challenge experiments in rhesus macaques following vaccination with a CMV-TB vaccine construct ^48^ and in mice following vaccination with the ΔMosR and ΔechA7 vaccine concepts ^64^ or the ΔSigH vaccine concept ^124^, all live, attenuated *Mtb* vaccines. If ongoing experiments continue to demonstrate sterilizing or near-sterilizing immunity with these or other vaccine concepts, this would suggest that a “limited dynamic range” of murine immunity was not the explanation for the limited degree of *Mtb* suppression noted with other vaccine candidates. The location and timing of the vaccine-induced immune response to *Mtb* (i.e. developing an *Mtb*-specific immune response in the lungs within days of initial infection, rather than weeks) may be the keys to a successful vaccination strategy against TB.

### e. Mucosal immunisation against TB

An inhaled TB vaccine makes intuitive and logistical sense for a number of reasons ^125^. Primarily, an inhaled vaccine mimics the route of *Mtb* infection, and offers the potential for generating potent and durable mucosal immune responses and stimulating specialized, peri-bronchial lymphoid tissue that may be critical in controlling TB. Additionally, BCG, administered through the universally used ID route does not reliably protect against pulmonary TB. From a logistical perspective, inhalation represents a common route of drug delivery which is safe, feasible, needle-free and pain-free.

In a phase 1 clinical trial assessing the safety and immunogenicity of 1×10 ^7^ plaque-forming units (pfu) of the MVA85A TB vaccine administered via inhalation or ID in 22 BCG-vaccinated adults, Ag85A-specific CD4+ T-cell responses in fluid obtained via bronchioalveolar lavage (BAL) were found to be stronger after AE than ID administration ^31^. Systemic Ag85A CD4+ T-cell responses were at least as strong after AE vaccination compared to ID administration. Additionally, no systemic anti-MVA vector IgG, IgA or IgM responses were detected following aerosol, but did develop following ID vaccination.

An important question to address when contemplating AE administration of TB vaccines is the safety of this administration strategy in persons with LTBI. This issue is being addressed in a phase 1 study of 15 persons with LTBI. Initial results from the first two patients enrolled have not raised safety concerns thus far.

Additional studies of TB vaccines administered via the AE route are due to begin soon, including a first-in-human study comparing AE and ID administered ChAdOx1.85 (University of Lausanne, Switzerland) and aerosolized Ad585B (McMaster University, Canada). A clinical trial assessing the safety and immunogenicity of increasing doses of BCG administered via the AE route based on encouraging protection and immunogenicity data in rhesus macaques, is ongoing, with no apparent safety issues raised and with encouraging, early immunogenicity data. It will be important to expand AE vaccination studies to sites endemic for TB to fully assess the safety, immunogenicity and efficacy of this vaccine administration strategy.

### f. Alternative routes of BCG immunization

An effective vaccine against TB will likely need to 1) stimulate an effective and persistent T-cell response against multiple antigens; and 2) generate this response in the lung, the site of initial *Mtb* infection and TB-associated pathogenesis.

Live, attenuated vaccines are an attractive vaccine strategy when targeting infections that are comprised of thousands of potential antigens and whose control depends on an effective and persistent T-cell response at tissue sites, such as malaria, leishmania and tuberculosis. Accordingly, live attenuated vaccines are an effective approach for generating T-cell responses of high magnitude and antigenic breadth compared to other vaccine platforms. Additionally, live or live-attenuated vaccines can stimulate T-cell responses in locations critical to inducing protection depending on the route of administration, thereby providing a rapid immune response at the time of infection. For malaria, intravenous but not subcutaneous immunization of a live, attenuated whole sporozoite vaccine was shown to induce a high frequency of antigen specific T-cells in the livers of NHPs. Also, attenuated sporozoites administered IV but not SC could protect humans against experimental controlled challenge ^126^. These data highlight the importance of the route of immunization on an infection requiring tissue resident T cells to multiple antigens.

For TB, the route of vaccine administration also influences the sites of T-cell priming, and the location and strength of where tissue resident T-cells develop. ID or IM vaccination is the route by which BCG and all other TB vaccine candidates in phase 2 or phase 3 clinical trials are administered respectively. These routes primarily result in T-cell priming in local draining lymph nodes, a moderate circulating T memory (T _mem_) response and a weak lung-resident effector (T _eff_) memory response, not an optimal situation when a rapid, lung-based T-cell response is needed for protection. Vaccines administered via the AE route result in local T-cell priming in the lung with a weak peripheral response ^124, 127, 128^. Combining ID or IM administration with AE administration theoretically represents a strategy by which both lung T _eff_ memory cells, in combination with circulating T _mem_, may be generated. With regard to AE delivery of vaccines, a series of NHP experiments with recombinant adenoviral-vectored TB vaccines encoding 2–4 antigens delivered via AE, generated robust T cell immunity in the lung, but did not result in greater protection against TB disease than with BCG given ID ^129^. These data suggest that there was either lack of antigenic breadth presented by the vectors, poor quality of the virally induced responses, or unfavorable innate imprinting by the viral vectors associated with increased risk of *Mtb* infection. Based on the results of clinical trials with subunit vaccines given by IM route and the NHP data by the AE route, data suggest that vaccines with greater antigenic breadth and an alternative route of immunization may be optimal.

Vaccines administered IV result in T-cell priming in the spleen, and strong lung-resident T _eff _memory and peripheral T _mem_ responses. Previous *Mtb* challenge experiments in NHPs receiving IV BCG administration resulted in greater protection than NHPs receiving either ID or a combination of ID and intra-tracheal BCG ^30, 130, 131^. To directly assess the effect of various vaccine administration routes of a vaccine presenting a wide breadth of antigens, rhesus challenge experiments were conducted with BCG administered by ID, AE, IV and ID + AE, with a low-dose (15 CFU) challenge 6 months after immunization (BCG doses: ID – 5×10 ^5^; AE – 5×10 ^7^; IV – 5×10 ^7^) (Robert Seder, personal communication). Initial immunogenicity results from BAL samples demonstrated that IV BCG resulted in a significant increase in the frequency of TB-specific CD4+ T-cells, and CD8+ T-cells compared to the other administration routes. Additionally, IV BCG strikingly altered the proportion of T-cells and macrophages in BAL samples. The extent to which IV BCG actually persists in the lung, and the degree of protection against *Mtb* mediated by administration of BCG via the IV route, is being assessed.

### g. Progress in clinical use of a controlled human infection model (CHIM) for vaccine testing

Controlled human infection models (CHIMs) have proven useful in vaccine development efforts, including vaccines for malaria, influenza and *Salmonella typhi*. Development of a CHIM for TB vaccine development would represent a major step forward, given the uncertain predictive value of preclinical animal models, the lack of a validated immune correlate of protection and the value of detecting a signal of vaccine protection in the host species. A CHIM to support TB vaccine development could be used for vaccine selection as well as for immunobiology studies to inform basic knowledge gaps.

Developing a TB vaccine CHIM is challenging task given that virulent *Mtb* cannot be used in the challenge model for obvious ethical reasons. Accordingly, a major question facing TB vaccine CHIM developers is whether *Mtb* can be manipulated to be safe enough to administer to volunteers. An additional question is whether BCG could be used either as the challenge organism or at least as an agent that would permit further clinical development of the clinical challenge model.

There are two key elements to developing a human challenge strain of *Mtb* for a CHIM: 1) developing a control system to elicit bacterial death; and 2) developing a system to detect viable *Mtb* in the days and weeks following challenge. When developing a system to control *Mtb* death, consideration must be given to the need to permit *Mtb* survival for multiple generations to permit an assessment of the effect of the vaccine on *Mtb* survival. At the end of the experiment, the killing control system must be able to eliminate all bacteria without relying on a lengthy course of antimycobacterial agents, and without the possibility of clinical relapse. Currently, three potential viability control systems are being assessed. The first strategy relies on the degradation of non-canonical amino acids upon which *Mtb* auxotrophs are dependent for survival. Another approach is the insertion of “kill switches” into the *Mtb* genome, which are inducible through the administration of exogenous molecules, such as tetracycline, that induce a gene that codes for a mycobacterium-directed toxin. Thirdly, a strategy involving protein degradation targeting is under investigation. It is possible that more than one of these approaches could be used in combination to improve safety.

Detection systems for viable *Mtb* will need to measure the levels of bacterial load in the lungs without relying on a CFU count. Moreover, the detection strategy must be non- or minimally invasive. Detection systems being assessed for a TB CHIM include skin detection of fluorescent proteins, serum detection of metabolites engineered to be expressed by the *Mtb* challenge strain, and breath detection of engineered volatile organic compounds.

Many questions regarding a CHIM for TB vaccine development remain to be answered, questions that will confront both CHIM developers and regulators tasked with assuring the safety of the mycobacterial control and detection strategies ultimately selected for the initial TB CHIM. Safety is paramount; questions regarding the confidence in the level of safeguards built into the *Mtb* challenge strain, and the potential for reversion to a virulent state, will need to be addressed. The duration of survival of a CHIM *Mtb* strain necessary to permit an adequate assessment of potential vaccine protection, and the level of sensitivity of detection that would need to be built into the system to permit these assessments remains unclear. Whether or not a vaccine effect can actually be detected via a CHIM strategy, and, if so, the types of TB vaccine for which this approach would be relevant, also remains unclear. Finally, the likelihood of regulatory acceptance of any *Mtb* CHIM is not yet known.

Given these uncertainties, a different CHIM strategy, based on a human intradermal (ID) BCG challenge, is being developed in parallel to CHIMs based on intrapulmonary *Mtb* administration. The rationale behind the ID BCG CHIM is based on the theory that an effective vaccine against BCG also should protect against *Mtb*. BCG vaccination has been found to suppress growth of an ID BCG challenge in mice, cattle and NHPs ^22, 132, 133^. A pilot BCG challenge study has been conducted in which volunteers freshly vaccinated with BCG, and those who were BCG naïve, were administered BCG ID, with punch biopsies obtained at 1, 2 or 4 weeks following ID BCG administration ^23^. PCR and culture assessments of the biopsy specimens demonstrated a small but significant degree of protection. A similar study, in which MVA85A and BCG + MVA85A was assessed in addition to persons vaccinated with BCG alone, also revealed a statistically significant reduction of ID BCG as determined by culture and PCR of skin punch biopsies in persons receiving BCG or BCG + MVA85A as compared to placebo or MVA85A alone ^134^.

Efforts are currently underway to develop a human AE BCG challenge model. Key issues in this effort are the safety and tolerability of BCG administered via AE and the ability to recover BCG in the bronchoalveolar lavage fluid (BALF). No safety concerns have been reported from the study to date. Early assessments suggest that the extent to which BCG can be recovered in BALF appears to be low, which may indicate the need to administer higher BCG doses.

In summary, work is ongoing to develop an attenuated, labelled *Mtb* strain for use in a human challenge study. An *Mtb* CHIM is approximately 3–5 years away from clinical use, and regulatory discussions regarding its development are ongoing. Work is progressing on ID and AE BCG challenge models. Overall, a CHIM to assess TB vaccine candidates is considered feasible. Ultimately, however, any CHIM developed for this purpose will require validation against field efficacy trials.

## 6. Perspectives from funders and global health organizations working on TB vaccines

### a. Perspectives from the Bill and Melinda Gates Foundation

The BMGF represents the largest global funder of research and development efforts specifically directed at TB vaccine development. The BMGF approaches TB vaccine development from the perspective of four strategic objectives:

1. To understand the natural immune response associated with protection against infection and disease: Efforts are underway to better understand the workings of the granuloma, the histopathologic signature of *Mtb* infection and disease ^135^. Unexpected diversity of granulomas has been shown in infected NHPs, even within the same animal: some granulomas are sterile while others appear permissive for *Mtb* survival ^21, 136, 137^. Ongoing research seeks to understand the immunological mechanisms underlying permissive and non-permissive granulomas, and correlating results with those found in human granulomas obtained from surgical resections. Prospective cohort studies focus on outlier populations, such as persons who do not develop *Mtb* infection despite presumed prolonged exposure to household contacts with active TB disease. Differences in host control between these persons and individuals who become infected are being investigated. Smaller experimental medicine studies will address specific immunological hypotheses. Knowledge generated from these efforts should inform rational vaccine design.

2. To develop new vaccine concepts that exploit immunological diversity: The focus is on vaccination approaches that target unconventional (non-Th1) immunity ^84^ and /or target the lung directly (e.g., mucosal vaccination) ^125^. In addition to delineating immunity that occurs in the natural infection setting, induction of “unnatural immunity,” will also be explored, i.e., immune responses not induced during natural infection and disease. An example of a vaccine that fits this strategy is the CMV-vectored TB vaccine, being developed by Louis Picker and colleagues at the Oregon Health Sciences University (OHSU), with major support from the BMGF ^59^. Other examples of unconventional immunity targeted include those mediated by antibodies or B cells ^72^, CD-1 restricted T cells ^138^, and MAIT cells ^139^.

3. To develop improved tools and infrastructure to support an efficient, iterative process to test vaccine concepts: The BMGF continues to refine the paradigm for testing vaccine candidates, to more efficiently test candidates in human studies, to use improved animal models for up selection of candidates, and to improve learning along the experimental pathway. The BMGF supports development of improved NHP models, primarily, in support of this goal.

4. To foster greater innovation, collaboration and coordination within the TB vaccine landscape: In support of this the BMGF has created the Collaboration for TB Vaccine Discovery (CTVD), an international network of scientists and experts dedicated to fostering innovation, cooperation, and collaboration in the up-stream TB vaccine discovery space. Further activities include improved alignment with other funders and exploring global portfolio management for clinical testing of vaccines through the use of stage gating.

TB vaccine discovery is in an early stage, lagging drug discovery efforts by at least 10 years. Accordingly, the BMGF is putting increased focused on the upstream space with the intent of learning more about *Mtb* pathogenesis, and the immune responses necessary to control TB disease and prevent established *Mtb* infection. This information will help guide more rational and efficient TB vaccine development in the future.

### b. The Global TB Vaccine Partnership

The Global TB Vaccine Partnership (GTBVP) is a five-year-old initiative which is comprised of the leading organizations conducting and supporting TB vaccine research and development. The goals of the GTBVP are to enhance communication between key members of the TB vaccine R&D community, as well as between members of the community and the public at large—particularly public and private decision-makers responsible for allocating resources in support of critical global health issues – in an effort to attract new funding to this under-resourced initiative. The GTBVP is made up of a leadership forum, a technical advisory group and a communications group. Under the direction of the GTBVP, a document describing the current state of the TB vaccine development effort, as well as future directions in TB vaccine R&D, is being drafted. Current plans call for this document to be revised every two years to reflect the rapid changes occurring in this dynamic field.

### c. Aeras/International AIDS Vaccine Initiative (IAVI)

Aeras, for years a nonprofit organization dedicated to the development of TB vaccines, had its assets transferred to the International AIDS Vaccine Initiative as of October 2018. Aeras/IAVI representatives stressed that TB vaccine R&D efforts are extremely resource constrained, with the burden falling on a few donors. The human impact of the disease in terms of suffering and death, along with the $19.2 billion annual cost in treatment, care and loss of productivity, is far out of balance with the small and inadequate investment currently being made in TB vaccine development and testing.

A lack of balance also exists in the nature of the partners involved in TB vaccine R&D, as support from non-profit organizations, such as the BMGF, and governments far outweigh the involvement of commercial entities such as established pharmaceutical companies and smaller biotech companies. Additionally, the shortage of funding has resulted in a lack of academic partners and new researchers entering the field. Moving forward, it will be important to create incentives for industry to increase activity in TB vaccine development initiatives.

Balance also needs to be achieved between support of “upstream” (basic research and discovery) and “downstream” (assessments of vaccine concepts and candidates in animal challenge models and human clinical trials) efforts. Increasingly, the scarce research dollars that exist are flowing upstream at the expense of support for downstream initiatives, particularly clinical trials. It will be critical to continue to move forward with animal challenge studies and clinical trials utilizing a diversity of vaccine candidates and vaccine strategies, with a commitment to challenging dogma. It will be important to recognize, however, that TB vaccine development is challenging, and many efforts will lead to failure. Failure must be accepted if success is to be achieved.

### d. European and Developing Countries Clinical Trials Partnership

The European and Developing Countries Clinical Trials Partnership (EDCTP) funds clinical research to accelerate the development of new or improved drugs, vaccines, microbicides and diagnostics against HIV/AIDS, tuberculosis and malaria as well as other poverty-related infectious diseases in sub-Saharan Africa, with a focus on phase 2 and 3 clinical trials. An important EDCTP goal is to help foster collaborative interactions within the TB vaccine development field. An example of this is the collaboration between TuBerculosis Vaccine Initiative (TBVI), Aeras, the European Commission (EC), BMGF and EDCTP in developing stage gate criteria, designed to promote an efficient and coordinated approach to managing the TB vaccine portfolio by selecting the best TB vaccine candidates and advancing them through development in an optimized and non-redundant fashion.

The EDCTP believes that it is imperative to continue conducting clinical trials of TB vaccine concepts and candidates. To further this effort, the EDCTP is contributing to the support of three clinical trials of TB vaccines in the upcoming years. These include 1) a pivotal, phase 3 study of VPM 1002 in African infants (conducted by Vakzine Project Management, Hannover, Germany and the Serum Institute of India, Pune, India); 2) a phase 2a dose-defining safety and immunogenicity study of MTBVAC in South African infants (sponsored by Biofabri, S.L.); and 3) a phase 2b PoR trial of H56/AS01E in adolescents and adults in South Africa and Tanzania (sponsored by the Statens Serum Institute and the International AIDS Vaccine Initiative [IAVI]). Additionally, the EDCTP will help support development of a human challenge model for TB, given the potential utility of such a model in driving the TB vaccine development effort forward.

### e. The Stop TB Partnership, working group on new TB vaccines

The Stop TB Partnership exists to support the WHO in its efforts to eradicate TB. Key goals of the Partnership are to strengthen the capacity and research literacy of advocates, and to strengthen the advocacy literacy of researchers. Developing advocates for TB vaccine development must represent an essential component of any strategy geared to increasing funding for TB vaccine R&D efforts.

### f. The Tuberculosis Vaccine Initiative

The TuBerculosis Vaccine Initiative (TBVI) is a non-profit foundation that facilitates the discovery and development of new, safe and effective TB vaccines that are accessible and affordable for all people. As a Product Development Partnership (PDP), TBVI integrates, translates and prioritises R&D efforts to discover and develop new TB vaccines and biomarkers for global use. TBVI provides essential services that support the R&D efforts of its consortium partners—50 partners from academia, research institutes and private industry in the TB vaccine field. A realistic strategy for TB vaccine development includes support for both upstream research and downstream clinical trials. It is imperative that a robust, healthy pipeline of TB vaccine concepts and candidates be maintained if the field is to move forward. The stage gating initiative, designed to encourage optimal management of the global TB vaccine portfolio, represents an important initiative in furthering this goal.

### g. Proposed stage gate criteria for TB vaccines

Projects stages, stage gates, and stage-gate criteria are a suite of tools that allow effective management of large R&D programs by sponsors and funders. An R&D program can be divided into segments referred to as stages, wherein multiple mostly related activities are conducted in parallel. Stage gates are defined as check points that separate the stages and the stage-gate criteria constitute a set of objective, pre-defined data targets upon which continuation of the project into the next stage is decided. A stage-gating tool has been in use by TBVI and AERAS since 2010 ^140^.

The BMGF is now funding a new joint TBVI-Aeras effort to revise the general TB vaccine stage-gate criteria that will aid vaccine researchers and developers as well as develop more specific stage- gating criteria based on vaccine target populations and/or vaccine indications.

The stage-gate criteria will be revisited biannually to ensure that the latest knowledge and developments relevant to the development of TB vaccines are included. The development process will include consultation with stakeholders participating in TB vaccine development.

Four different indications for TB vaccines will be addressed under the stage-gate criteria: 1) prevention of TB disease in pediatric, adolescent and adult/elderly populations; 2) BCG replacement in infants; 3) prevention of TB disease recurrence; and 4) immunotherapy of active TB disease. Stage gating criteria for these indications may differ in pre-clinical immunogenicity, efficacy and safety requirements; in clinical development strategy, including safety, immunogenicity and efficacy targets, and target populations; and in regulatory and marketing targets. Commonalities between indications will be found in process and manufacturing criteria, and well as in the overall precut characteristics. These differences and commonalities ultimately will be reflected in the stage gating criteria. Efforts towards the finalization of the stage gating document are underway.

## 7. Conclusions

As the TB vaccine development field moves forward, it will be important to educate potential funders as to the barriers faced in conducting clinical trials of TB vaccines and to clearly frame the specific goals of these trials. The WHO holds an important responsibility for bringing researchers and funders together in support of needed efforts.

The WHO has provided excellent leadership through its introduction of preferred product criteria (PPC) for TB vaccine development ^5^. Importantly, the criteria appear to be flexible enough to keep open many directions in TB vaccine research and avoiding the trap of focusing on the wrong strategic priorities. While the WHO is presenting different PPCs for TB vaccines targeting infants and adolescents/adults, respectively, it will be important to model the effect of using adult and infant vaccines together to end the TB epidemic.

This is a critically important juncture for TB vaccine development. Results from the recently concluded BCG revaccination study in South African adolescents at high risk of acquiring *Mtb* infection have reemphasized the potential promise of looking at this old vaccine in new ways ^12^. M72/AS01E candidate vaccine results, published after the meeting reported here, constitute a major encouragement for the field, and argue in favor of strengthening global health stakeholder engagement and funding for further TB vaccine R&D efforts ^141^. Ongoing, robust support for TB vaccine development will be critical to ensuring that a TB vaccine is available to help end the global scourge of tuberculosis.

## Data availability

No data are associated with this article.
