# Supplementary material for: Research and development of new tuberculosis vaccines: a review
Source: F1000Res. 2019 Feb 24;7:1732. Originally published 2018 Nov 1. [Version 2] doi: 10.12688/f1000research.16521.2 (PMC6305224; doi:10.12688/f1000research.16521.2)
Supplement: Peer review history

# Peer review: Reviewer response for version 1

**DOI:** 10.5256/f1000research.18057.r41389

**Published:** 2019-01-14

**Authors:** Salil Bhargava, Priyanka Chaskar

**Affiliations:** 1 Dept. of Chest & TB, MGM Medical College Indore, Indore, India; 2 Department of Microbiology, MGM Medical College, Indore, India

The review article has covered the problem statement, the need for the development of new vaccines and the newer vaccines in phase 2 and 3 very well. This article covers the newer platform i.e. mRNA and new viral vector hCMV for vaccine delivery. The authors have also elaborated on immune mechanisms stimulated in response to Mtb including HLA E, CD1a, antibody response in addition to MHC I and II responses. These mechanisms are targeted by newer vaccine candidates to generate an immune response in adults and adolescents.

 The review also covers the use of advanced assessment techniques, including transcriptomics, proteomics and metabolomics for identifying newer biomarkers for identifying active disease and can further be used to assess vaccine efficacy and safety. The article also highlights alternative routes of immunization and discusses BCG revaccination status.

 However, it would be helpful if the following points are discussed in the review:

1. Role of DNA vaccines (Tian *et al.*, 2018 ^1^ and Bruffaerts *et al.*, 2014 ^2^).
2. 231 subcultures in place of 230 subcultures over 13 years to develop BCG on page 7 (Norouzi *et al.*, 2012 ^3^).

We have read this submission. We believe that we have an appropriate level of expertise to confirm that it is of an acceptable scientific standard.

## References

[1] TianMZhouZTanSFanXLiLUllahN: Formulation in DDA-MPLA-TDB Liposome Enhances the Immunogenicity and Protective Efficacy of a DNA Vaccine against Mycobacterium tuberculosis Infection. *Front Immunol*.2018;9: 10.3389/fimmu.2018.00310 310 10.3389/fimmu.2018.00310 29535714PMC5835323

[2] BruffaertsNHuygenKRomanoM: DNA vaccines against tuberculosis. *Expert Opin Biol Ther*.2014;14(12) : 10.1517/14712598.2014.951630 1801-13 10.1517/14712598.2014.951630 25145964

[3] NorouziSAghamohammadiAMamishiSRosenzweigSDRezaeiN: Bacillus Calmette-Guérin (BCG) complications associated with primary immunodeficiency diseases. *J Infect*.2012;64(6) : 10.1016/j.jinf.2012.03.012 543-54 10.1016/j.jinf.2012.03.012 22430715PMC4792288

# Peer review: Reviewer response for version 1

**DOI:** 10.5256/f1000research.18057.r41392

**Published:** 2018-12-19

**Authors:** Roberto Zenteno-Cuevas

**Affiliations:** 1 Instituto de Salud Pública, Universidad Veracruzana, Xalapa, Verazruz, Mexico

The aim of this review was to show the state of the art tuberculosis vaccine development, also describing the major problems related with this issue viewed from the public health, the financial, and governance. The major difficulties and pitfalls related within these aspects are properly addressed, and the final conclusions are clear and well presented.

 Also, it has to be recognized the value of the inclusion of new elements in this kind of review, such as the short review of the WHO global TB program, the value proposition for TB vaccines, the mathematical modelling techniques and its impact in the evaluation of new vaccines, bio-signatures, biomarkers and “omic” sciences, and the perspectives of the funders for the development of vaccines.

 I am certain that this review will be of great interest for those researchers related to tuberculosis vaccines and all people interested in this thematic. 

 In conclusion, this review could be suitable for indexing in the actual condition, no modifications or corrections are suggested.

 Great job, congratulations to the authors!

I have read this submission. I believe that I have an appropriate level of expertise to confirm that it is of an acceptable scientific standard.

# Peer review: Reviewer response for version 1

**DOI:** 10.5256/f1000research.18057.r41391

**Published:** 2018-12-17

**Authors:** Stefan H.E. Kaufmann

**Affiliations:** 1 Department of Immunology, Max Planck Institute for Infection Biology, Berlin, Germany

The review provides a comprehensive summary of a WHO meeting on vaccine development and also includes reference to some relevant publications published afterwards. Unfortunately, the review is somehow unbalanced in several instances (see below) and in several cases uses specific immunologic details which are insufficiently explained and therefore may be hard to understand for a non-expert reader (see below). Moreover, in several cases, the article was written in a sloppy way (see below).

 Some comments (not at all comprehensive):

1. P. 4, left column: Compare writing of US dollar here and p. 4, right column, 2 ^nd^ paragraph. Be consistent.
2. P. 4, right column, 1 ^st^ paragraph, 2 ^nd^ sentence: It doesn’t make sense to me the way it is written.
3. P. 7, 2 ^nd^ paragraph: What is a truly effective vaccine? A vaccine which protects everybody lifelong? Or a vaccine which protects everybody for a certain time? Or some for a certain time?
4. P. 8, left column, 1 ^st^ and 2 ^nd^ paragraph: Need references
5. P. 8, right column, 2 ^nd^ paragraph: It is likely that BCG does not protect against bladder cancer, but is a therapeutic immunomodulator.
6. P. 8, right column, 2 ^nd^ last paragraph: VPM is being tested at different sites in Sub-Saharan Africa, not just South Africa.
7. P. 9, left column, 1 ^st^ paragraph: What does the discussion about epitopes really mean for vaccine efficacy? Does this assume that every epitope is equally important (dominant vs. subdominant)?
8. Discussion of different vaccines: a) This whole section from page 8 to 12 to this reviewer appears quite imbalanced both in length and details of individual candidates. B) Wherever possible, registration numbers of trials should be included. C) For some candidates a very detailed description is being given which may be understandable for those in the field, but may be less so for outsiders.
9. P. 30, 3 ^rd^ paragraph: Another example for highly specific details which are not explained and therefore may be difficult to understand since text mentions all the different presentation molecules for T cells (CD1, MR1 etc.) without further explanation about their relative importance (e.g. compared to canonical MHC molecules).
10. P. 13, right column, 2 ^nd^ last paragraph: Role of antibodies in animal models: To this reviewer, several experimental animal models and experiments argue against a role for B cells. This topic would need to be discussed more carefully (this reviewer agrees that antibodies likely play a role in human TB).
11. P. 15, right column, end of 2 ^nd^ paragraph: A kind hint: The paper is now out (Weiner et al., Nat Commun, 2018)
12. P. 16, right column, 2 ^nd^ paragraph, Ref. 110: incorrect. Best to provide relevant website?
13. P. 19: Discussion about memory T cells could benefit from the short description of the different subtypes and their biological function.
14. P. 19, right column, 2 ^nd^ paragraph: Is there a reference or is this a personal communication (from whom)?
15. P. 20, left column, 2 ^nd^ paragraph: Is there a reference or is this a personal communication (from whom)?
16. P. 21, left column, last paragraph: Is there a website for GTBVP?
17. P. 21, right column: paragraphs about Aeras: It might be worth to mention that this entity does not exist anymore (reviewer is aware that this happened after the meeting). Text has no direct relationship with Aeras, but rather describes strategies in general.
18. P. 21, right column: EDCTP paragraphs: It would be helpful to mention TB vaccine trials supported by EDCTP (several!).

I have read this submission. I believe that I have an appropriate level of expertise to confirm that it is of an acceptable scientific standard, however I have significant reservations, as outlined above.

# Peer review: Reviewer response for version 1

**DOI:** 10.5256/f1000research.18057.r40107

**Published:** 2018-11-26

**Authors:** Gerald Voss

**Affiliations:** 1 SciPeo Sprl, Lelystad, Belgium; 2 Tuberculosis Vaccine Initiative (TBVI), Lelystad, The Netherlands

This review article provides a comprehensive and detailed summary of a WHO consultation on TB vaccine development. It is well structured, clearly written and based on the presentations during the consultation.

 The article describes the need for new TB vaccines and provides an overview of the current clinical pipeline as well as considerations and modeling for anticipated impact. Subsequently, the different indications and populations that would benefit from new TB vaccines are described, notably BCG replacement and vaccines for adolescent and adult populations. An overview of new vaccine platforms, for example CMV, mRNA and whole cell vaccines, is provided. Then, the importance and usefulness of models for TB vaccine development, from rodents to controlled human challenge studies, is laid out. The final section provides perspectives from the main funders and other stakeholders in the field.

 Importantly, new developments, including clinical data on BCG revaccination and the M72/AS01E TB vaccine candidate have been incorporated to reflect the major recent advances in the field. This review is a comprehensive and useful reference for the current state of TB vaccine development.

I have read this submission. I believe that I have an appropriate level of expertise to confirm that it is of an acceptable scientific standard.
